# Single‐Cell Nucleus Extraction with Cellular Indexing

**DOI:** 10.1002/advs.202514883

**Published:** 2025-09-29

**Authors:** Trinh Lam, Ana Esmeralda Gomez Martinez, Alison Su, Anna Fomitcheva‐Khartchenko, Xin Wang, Md Nazibul Islam, Paul Lum, Amy Elizabeth Herr

**Affiliations:** ^1^ Department of Bioengineering University of California Berkeley Berkeley CA 94720 USA; ^2^ Chan Zuckerberg Biohub San Francisco San Francisco CA 94158 USA; ^3^ The California Institute for Quantitative Biosciences at UC Berkeley Berkeley USA

**Keywords:** hydrogels, microfluidics, multiomics, organelles, proteomics, single‐cell

## Abstract

Bulk organelle‑fractionation masks cell‑to‑cell heterogeneity, and existing microfluidic methods cannot reliably reconnect each isolated organelle to its parent cell, an essential capability for multiomics readouts. VacTrap, a high‐throughput microfluidic device that isolates and spatially indexes single nuclei from mammalian cells is developed. The VacTrap device consisted of three aligned layers: 1) a Bis‐gel microwells layer with a “trapdoor” (BAC‐gel) base, fabricated atop a through‐hole glass slide; 2) a polydimethylsiloxane (PDMS) microwell layer to receive transferred nuclei; and 3) a vacuum manifold. VacTrap operation begins with cell cytoplasmic lysis using differential detergent fractionation (DDF) to release intact nuclei into the Bis‐gel microwells, while cytoplasmic proteins are electrophoresed into the Bis‐gel layer. Subsequent addition of dithiothreitol (DTT) and vacuum dissolves the trapdoors within 3–5 min, synchronously transferring nuclei into the PDMS microwells, achieving 98% efficiency across 80% of trapdoors. To verify fractionation of the cytoplasmic proteins from each cell nucleus, select protein targets are successfully detected by in situ immunoprobing in the archival Bis‐gel layer. To verify the fractionation and collection of individual intact nuclei, the morphology analysis confirms preservation of the nuclear features. By introducing spatial indexing of nuclei back to the originating cell, VacTrap provides a robust, automated cell‐preparation platform for single‐cell multiomics applications.

## Graphical abstract

VacTrap is a multilayer, high‐throughput microfluidic system that isolates and spatially indexes single nuclei from hundreds of cells within minutes. Using dissolvable hydrogel trapdoors triggered by chemical and vacuum actuation, VacTrap synchronously transfers nuclei while preserving spatial links to parent cells. VacTrap enables rapid, automated nuclei preparation for multiomics assays, maintaining nuclear integrity and archiving cytoplasmic proteomes.

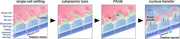

## 1 Introduction

Cells are composed of specialized organelles that each perform unique functions. Structural and functional assays rely on organelle isolation, wherein the integrity of the isolated organelles directly affects analysis accuracy, ultimately shaping our understanding of biology.^[^ ^1^ ^]^ Bulk organelle‐fractionation methods (e.g., density‐gradient centrifugation, immune‐isolation, free‐flow electrophoresis, detergent‐based chemical fractionation, enzymatic digestion) are labor‐intensive, designed for pooled cell suspensions and not suitable for sparingly available specimens, and offer low organelle‐recovery yields. Although suffering from these performance shortcomings, density‐gradient centrifugation remains widely used.^[^ ^2^, ^3^, ^4^ ^]^ Immuno‐isolation is constrained by the availability and quality of antibody probes specific to organelle surface proteins.^[^ ^5^ ^]^ Free‐flow electrophoresis separates cellular organelles^[^ ^6^, ^7^ ^]^ with low recovery purity and resolution.^[^ ^8^ ^]^ Detergent cocktails enrich specific cellular fractions, with each chemical component having a distinct solubilization efficiency.^[^ ^9^, ^10^ ^]^ Yet, enzymatic treatments are known to perturb cell‐cycle status, apoptosis, and structural alterations.^[^ ^11^, ^12^ ^]^ Overall, bulk organelle‐isolation methods require multiple steps requiring extensive manual handling and yield compromised purity and integrity of the isolated organelles, thus impacting functional analysis.

Microfluidic devices enhance precision in organelle isolation, which is particularly important in scarce starting specimens. Further, microfluidic organelle isolation approaches utilizing magnetic nanoparticles,^[^ ^13^ ^]^ immuno‐affinity,^[^ ^14^, ^15^ ^]^ flow‐based or channel structures,^[^ ^16^, ^17^, ^18^, ^19^ ^]^ digital microfluidics,^[^ ^20^, ^21^ ^]^ magnetophoretic‐based microfluidics,^[^ ^22^ ^]^ and devices structured to capture DNA^[^ ^23^, ^24^ ^]^ can overcome yield limitations and sample‐prep throughput. While precise, multi‐step process flows (e.g., on‐chip extraction, isolation, and off‐chip recovery) can be a source of organelle damage and yield loss.

Despite advances in precision organelle isolation, post‐isolation pooling remains commonplace. Pooling limits the applicability to multiomics questions that demand tracing of an organelle back to its originating cell. Indexing an isolated organelle to the originating cell forms a basis for understanding organelle‐derived heterogeneity that exists between cell types and among individual cells, even of the same type.^[^ ^25^ ^]^ In a related aspect of performance, the preservation of spatial information is increasingly sought, such as mapping an isolated organelle(s) back to the originating tissue context. Logically, mapping back to the single originating cell is also sought because functional links between biological processes can (and do) occur at the level of single cells.

An active area of organelle‐ and cellular‐level biology is the study of the nucleus as a coordinating – and typically the largest – cellular organelle. Microfluidic tools make single‐nucleus measurements possible. To analyze chromosomal DNA from single nuclei, Benítez et al. introduced a micropillar array and hydrodynamic flows to extract and stretch chromosomal DNA from ≈100 single mammalian cells per chip.^[^ ^26^ ^]^ Following these precise, in situ assays of chromosomal‐DNA stretching from a single cell, DNA was recovered and quantified off‐chip. Similarly, Wang et al. utilized microchannel geometries to isolate and stretch chromosomal DNA from 10–20 nuclei for subsequent DNA fluorescence in situ hybridization, with each signal traced back to its originating nucleus.^[^ ^27^ ^]^ While limited to assessing DNA damage, conventional agarose‐slab embedded and microwell‐based comet assays do allow researchers to assess DNA damage and map back to the originating cell.^[^ ^28^ ^]^ These existing techniques point to the promise for assessing other nuclear components – including proteins such as transcription factors – and mapping said measurements back to the originating cell and/or tissue context. The capability to index each nucleus to its cell of origin offers valuable advantages for multi‐omics workflows, which increasingly integrate genomic, transcriptomic, and proteomic data to probe cellular heterogeneity. Such single‐cell‐level analyses can reveal critical insights into patient‐specific disease mechanisms, potentially guiding precision therapies and personalized medicine.^[^ ^29^, ^30^, ^31^ ^]^ Nonetheless, challenges remain in data integration, computational complexity, and sample throughput. Microfluidic technology underpins emerging multi‐omics pipelines, with integrated microsystems designed to bridge the gap between mechanistic single‐cell studies and the broader goal of tailoring treatments to individual patient profiles.

With a focus on introducing tools for single‐cell resolution protein measurement, our group introduced a suite of single‐cell immunoblotting modalities designed using microwell‐isolated single cells, including single‐cell western blots.^[^ ^32^, ^33^, ^34^ ^]^ With an eye toward organelle‐biology and subcellular omics, we introduce tools for single‐nucleus isolation using microwell‐isolated mammalian cells subjected to differential detergent fractionation (DDF), a technique that employs a sequence of detergents with varying solubilization strengths to selectively extract and separate cellular components based on membrane properties.^[^ ^2^, ^35^ ^]^ In one example of a single‐nucleus resolution analysis made possible by combining microfluidic precision with DDF, we performed a single‐cell western blot of each cytoplasmic compartment and a distinct electrophoresis of each nuclear compartment for an array of cells. In a second example that extends on the assay just described, the western blot analysis of each single nucleus was swapped out with a PCR assay, allowing both cytoplasmic protein targets and nuclear DNA and RNA targets to be detected in the same originating cell.^[^ ^36^, ^37^, ^38^ ^]^ While both single‐nucleus precision assays are suitable for sparingly available starting specimens (<10 starting cells, for example, isolated circulating tumor cells, individual blastomeres comprising two‐ and four‐cell preimplantation murine embryos), sample and analysis throughput must be increased for applicability to larger‐cell‐number specimens.

Here, we introduce a single‐cell resolution nucleus isolation method incorporating a single‐cell isolation via a polyacrylamide microwell array that is optimized for nuclear isolation after DDF. We utilize microfluidic automation to enhance throughput while offering the capacity to isolate and then index individual nuclei back to each originating cell. In a multi‐layered, planar microfluidic device, individual cells are isolated by settling into an array of polyacrylamide gel (PAG) microwells, one cell per microwell. Perturbation, proteomics, or imaging analysis can be performed on intact cells in these top‐layer polyacrylamide gel microwells. To isolate and extract single nuclei for further analysis, each cell's cytoplasmic membrane is lysed using DDF, proteins are electrophoresed via polyacrylamide gel electrophoresis (PAGE), and one intact nucleus remains in each microwell. To concurrently transfer each nucleus to an aligned PDMS microwell situated below the PAG microwell, an interleaving layer of through‐holes filled with a dissolvable gel is actuated. These dissolvable “trapdoors” in the floor of each PAG microwell open when the dissolvable gel is exposed to reducing agents (e.g., dithiothreitol (DTT)) and suction is applied using an attached microfluidic vacuum manifold. Once the trapdoors are open, hundreds of nuclei are simultaneously transferred from the PAG microwell array to the PDMS microwell array, at one nucleus per microwell occupancy. Here, we detail the multi‐layer fluidic design, chemical and hydrodynamic control optimization, and resultant nucleus isolation and extraction performance of this single‐nucleus isolation and extraction technique.

## 2 Results and Discussion

**Figure 1:**
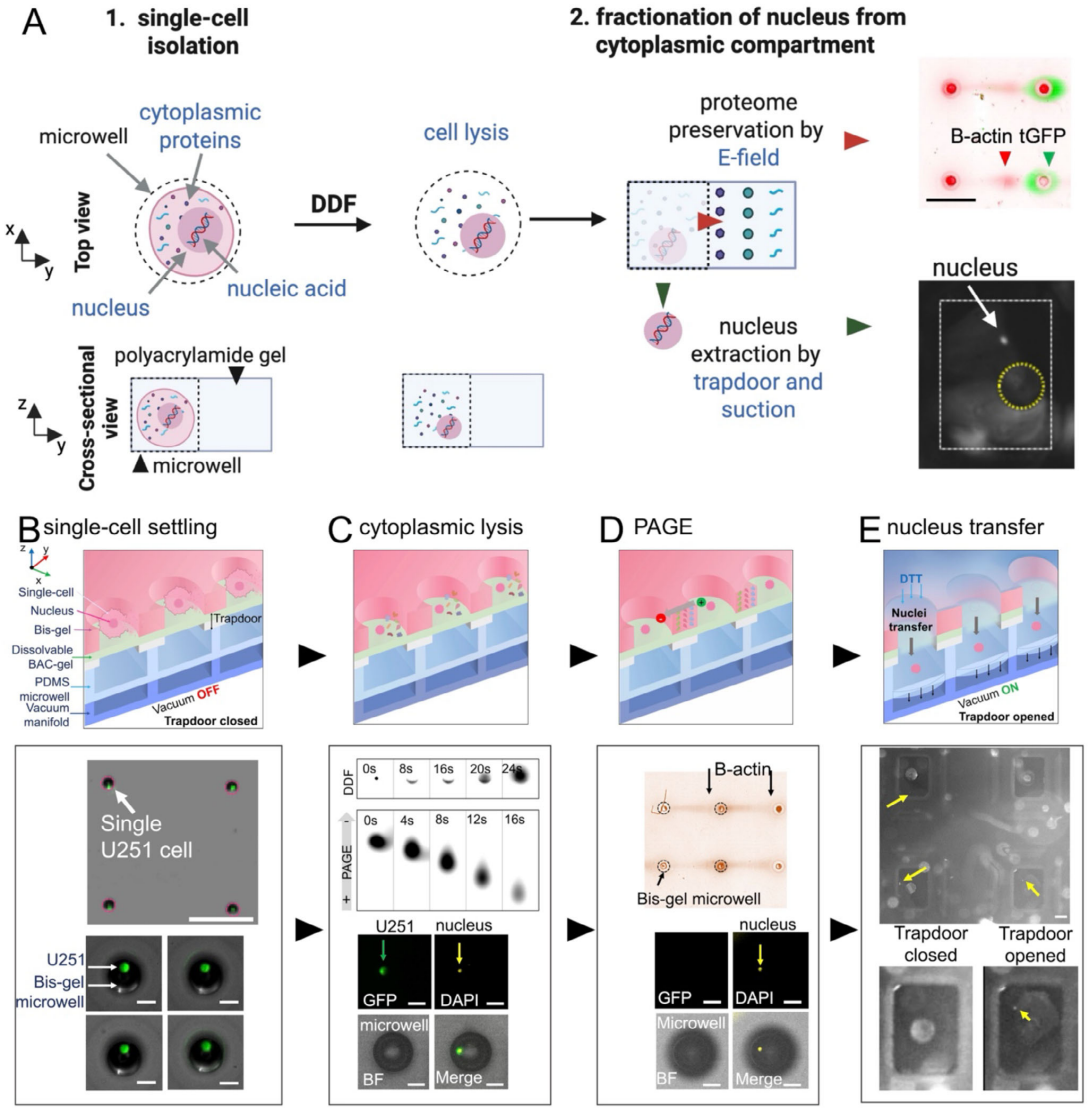
Integrated microfluidic VacTrap system fractionates and physically isolates cytoplasmic from nuclear compartments with single‐cell resolution. A) Conceptual schematic illustrates how VacTrap – a microwell‐based, multi‐layer device – acts to physically fractionate the cytoplasmic compartment from the nuclear compartment with single‐cell precision. B–E) Schematic and representative micrographs illustrate the VacTrap workflow (top: schematic; bottom: representative data). B) VacTrap consists of three aligned device layers: 1) a Bis‐gel microwell layer with a dissolvable BAC‐gel “trapdoor” base fabricated atop a through‐hole glass slide; 2) a PDMS microwell layer designed to receive nuclei; and 3) a vacuum manifold that activates the trapdoor. Single cells (U251‐tGFP) are gravity settled into the Bis‐gel microwells and whole‐cell imaging is conducted. C) To fractionate each cell's cytoplasmic compartment from its nuclear compartment, each cell's membrane is lysed by DDF lysis, thus solubilizing the cytoplasmic proteins and leaving the nucleus intact in the Bis‐gel microwells (yellow arrows). D) To physically isolate and archive cytoplasmic proteins, the solubilized cytoplasmic compartment is electrophoresed into the surrounding Bis‐gel layer for archiving while retaining each cell's index to its originating cell. E) To physically isolate and extract each intact nucleus, the trapdoors are exposed to DTT and vacuum, causing the trapdoor gel to dissolve, thereby allowing vacuum to actuate the physical transfer of each nucleus (yellow arrows) into PDMS microwells in the bottom layer while retaining indexing of each naked nucleus to its originating cell and archived cytoplasmic compartment. BF: bright field.

To advance organelle biology, subcellular omics, and multiomics, we introduce a microfluidic device that isolates nuclei from hundreds of individual mammalian cells for proteomic analysis via single‐cell western blot (scWB), while enabling precise handling, automation, and the ability to trace each nucleus back to its original cell (**Figure** **1**). By cytoplasmic‐specific lysing single cells to release nuclei for nucleic‐acid applications such as next‐generation sequencing (NGS), we also archive proteomic data from those same cells (Figure 1A). Using the NGS data, our platform facilitates target discovery and targeted analysis by leveraging sequencing insights to guide protein selection for immunoprobing. In this study, we used a multilayer microfluidic device (called VacTrap, for brevity), designed to perform a controlled, automated single nucleus preparation protocol, while archiving the proteomics of the single‐cell via single‐cell western blot. We report here on device design and fabrication, optimization of the chemical and mechanical functions of VacTrap (device and preparation protocol), performance of the single‐nucleus extraction system, and protein immunoblotting from the same single cell (Figure 1B–E).

### 2.1 Overall Design of the VacTrap Nucleus Isolation and Extraction Device

**Figure 2:**
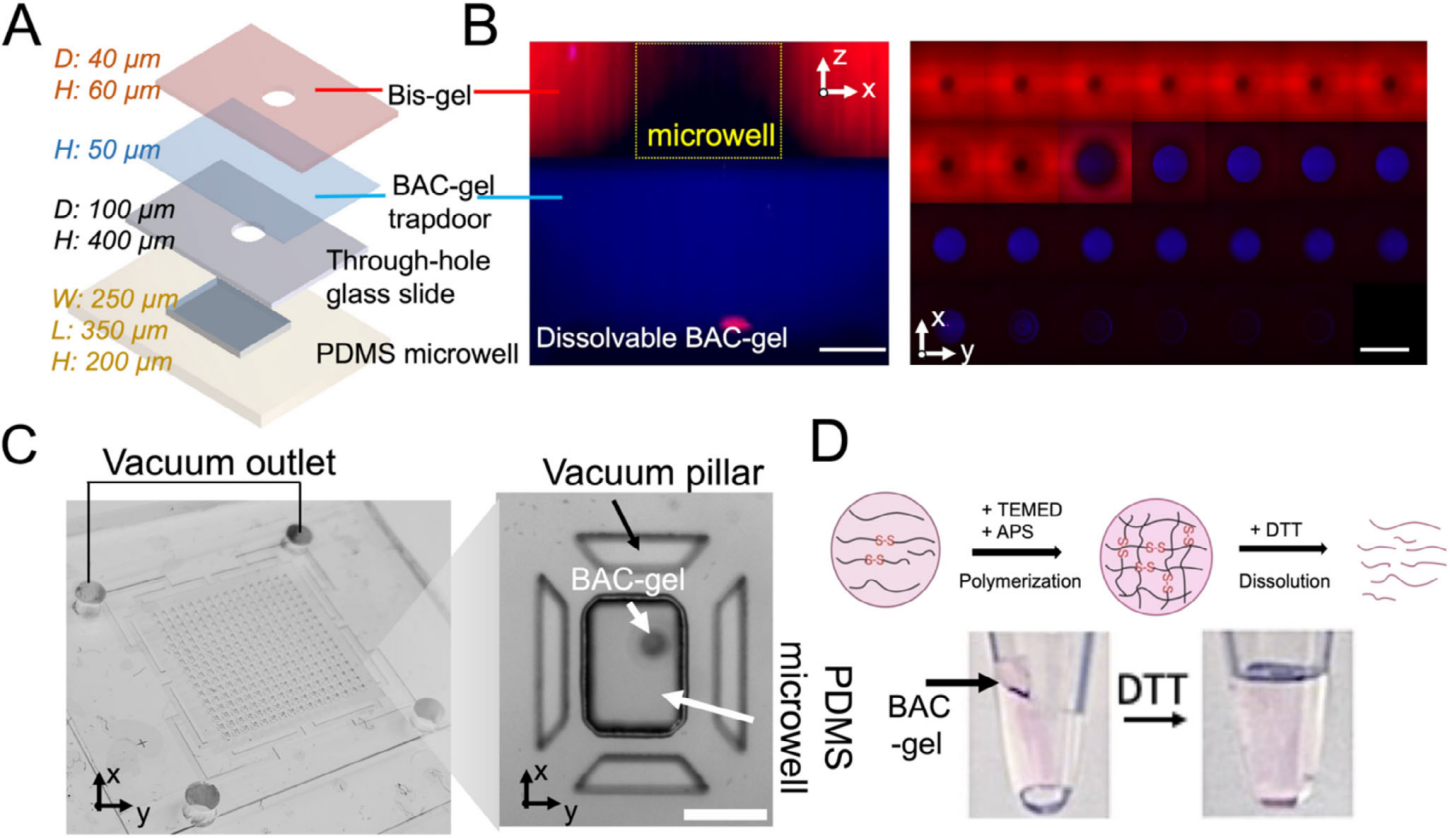
VacTrap uses a co‐planar multilayer microfluidic device with vacuum‐assisted (mechano) and DTT (chemico) actuation for single‐nucleus extraction with cellular indexing from a single cell. A) Detailed schematic of each layer of VacTrap. Sequential layers include a 60‐µm height and 40‐µm diameter Bis‐gel microwell (red), a 50‐µm height layer of dissolvable BAC‐gel (blue), 400‐µm‐thick through‐holes with a 100‐µm diameter through‐hole glass slide, and a 350 by 250 µm nucleus‐receiving PDMS microwell. B) Confocal imaging highlights both a cross‐sectional view (left; scale bar, 40 µm) and sequential confocal z‐sections (right; scale bar, 100 µm) of the Bis‐gel microwell with BAC‐gel trapdoor features, with each slice separated by 5 µm. (Left) 2D cross‐sectional reconstruction shows the Bis‐gel microwell stacked on the dissolvable BAC‐gel trapdoor. (Right) The series of confocal images used to generate the 2D cross‐section image. The Bis‐gel microwell was labeled with Rhodamine B methacrylate (red), and the BAC‐gel was labeled with FITC acrylate (blue). All images were acquired using a 40× water‐immersion Plan APO objective. C) Brightfield photograph of the assembled three‐layer VacTrap showing vacuum outlet ports. Micrograph inset shows a top–down view of the BAC gel to PDMS microwell pair stack, interleaving trapdoor layer, and vacuum trapezoid pillars to prevent deformation of the PDMS microwell while  suction force is applied. D) Schematic of the synthesis and dissolution mechanism of the dissolvable BAC‐gel used to create and open the trapdoor feature. Acrylamide monomers and BAC crosslinker undergo polymerization via C=C double bonds facilitated by ammonium persulfate (APS) and tetramethylethylenediamine (TEMED), resulting in the formation of a dissolvable polyacrylamide gel layer. Exposure to reducing agents (DTT) depolymerizes the disulfide‐crosslinked BAC‐gel due to the thiol–disulfide exchange reaction.

The VacTrap device design consists of three co‐planar layers (Figures 1B,2A): 1) a whole‐cell receiving layer which is the top, open‐fluidic, Bis‐gel layer stippled with an array of microwells each having a mechano‐chemically actuated “trapdoor” at the base of each microwell (**Figure** **2**B), and a vacuum manifold with trapezoid‐pillar array (Figure 2C).

**Figure 3:**
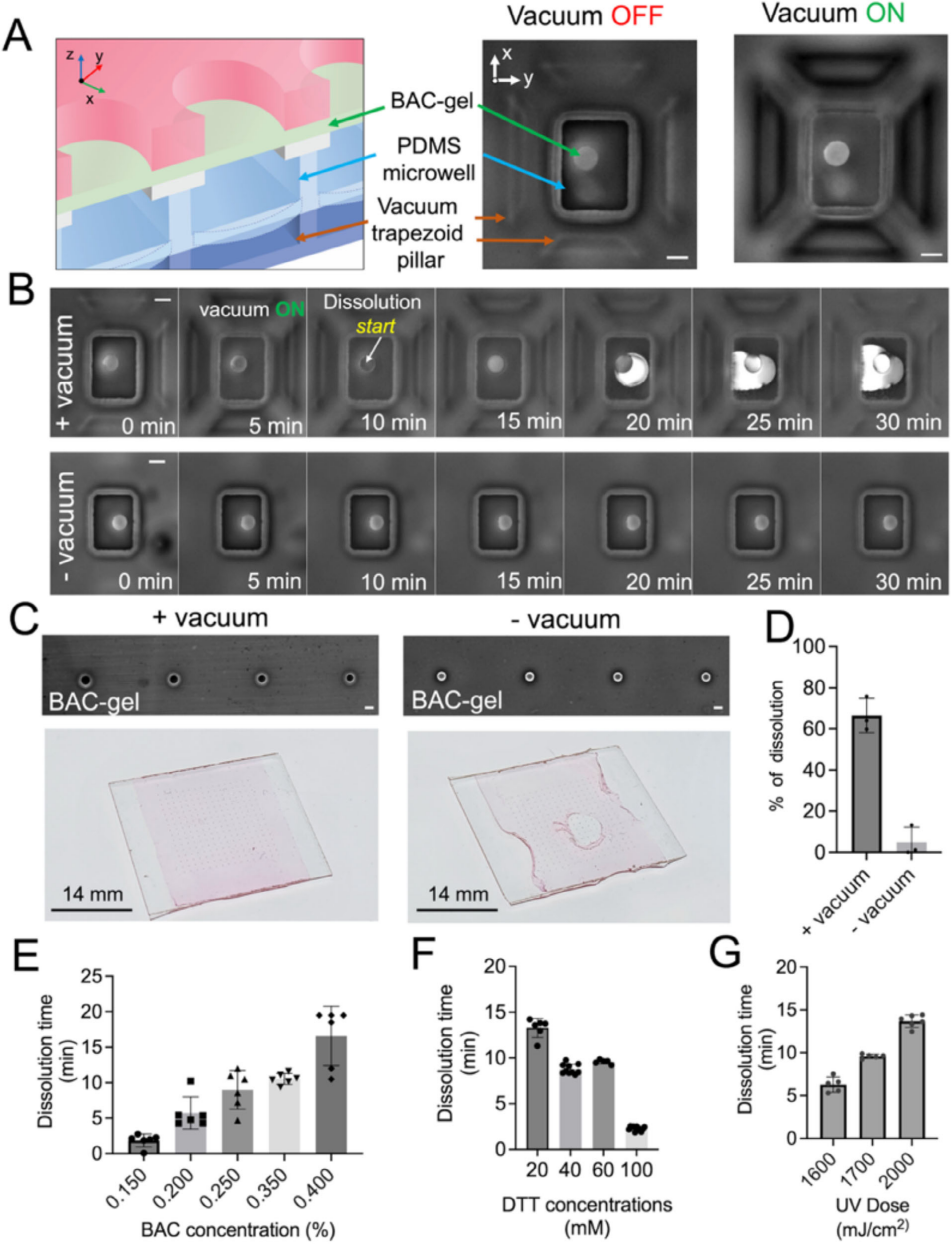
Chemico‐mechanical actuation dissolves and opens the trapdoor feature for the physical transfer of 384 single nuclei from the top‐layer Bis‐gel microwell to the bottom‐layer PDMS microwell in a VacTrap multilayer device. A) Fluorescence micrographs of pre‐ and post‐actuation of the trapdoor feature by an applied suction force levied by the vacuum manifold with trapezoid pillars. For visualization, the BAC‐gel is copolymerized with 0.2 mm FITC acrylate in all fluorescence images reported in this Figure. (Left) The 3D schematic shows the corresponding layer of VacTrap compared to the top‐view imaging in the right panel. (Right) Fluorescence micrographs show the structural integrity of trapezoidal pillars and PDMS microwell after suction is applied to the PDMS microwell by the vacuum manifold. Scale bar: 100 µm. B) Timelapse of fluorescence micrographs of the PDMS microwell and trapdoor feature report dissolution of the 0.2% BAC‐gel trapdoor with application of 40 mm DTT, observed with (+vacuum) and without (−vacuum) vacuum application. Fluorescence microscopy uses a FITC (488 nm) filter focusing on the BAC‐gel trapdoor. Scale bar: 100 µm. C) BAC‐gel trapdoor dissolution efficacy with and without vacuum applied (+vacuum, −vacuum). Micrograph imaging (Scale bar: 100 µm) shows the complete and specific dissolution of BAC‐gel around the through‐hole area, indicated by the loss of fluorescence in the through‐hole area. Without the vacuum, the dissolution is limited, and the fluorescence signal of the BAC‐gel remained around the through‐hole. Moreover, the non‐specific dissolution of the BAC‐gel causes gel damage and detachment due to loss of support beneath the Bis‐gel. D) BAC‐gel dissolution efficiency as determined by enumerating PDMS microwells exhibiting circumscribed FITC signal, indicative of successful BAC‐gel dissolution and physical transfer into the nuclei‐receiving PDMS microwell. E) Dissolution time of the BAC‐gel trapdoor as a function of BAC concentration with [DTT] = 100 mm. F) Dissolution time of the BAC‐gel trapdoor (0.2% BAC) as a function of applied DTT concentration. G) Dissolution time of the 0.2% BAC‐gel trapdoor with 40 mm DTT as a function of UV dose used for Bis‐gel microwell photopolymerization. Fixed acrylamide concentration of 6% w/v in E–G.

The trapdoor feature consists of a layer of chemically dissolvable BAC‐gel coated on a glass support slide with through‐holes, 2) a nucleus‐receiving layer molded with a PDMS microwell array, wherein each PDMS microwell is aligned to an upper Bis‐gel microwell and BAC‐gel trapdoor (Figure 2A), and 3) a vacuum manifold layer with trapezoid pillar to apply a suction force that, together with the application of DTT, a common redox agent, drives the simultaneous transfer of nuclei from the cell‐laden Bis‐gel microwells to the nucleus‐receiving PDMS microwells (Figures 1E,2C,D). After sedimentation and imaging of intact whole cells in the Bis‐gel microwells of the top whole‐cell receiving layer, nuclei are concurrently isolated from the cells prior to transferred to the nucleus‐receiving PDMS microwells. To isolate nuclei, we introduce a cytoplasmic lysis buffer that selectively lyses each cell's cytoplasmic membrane, leaving the nuclear membrane, and thus the nucleus, intact in each top‐layer Bis‐gel microwell^[^ ^34^ ^]^ (Figure 1B–E).

We selected three distinct materials for the whole‐cell and nucleus‐receiving microwell array layers: PAG (BAC‐gel and Bis‐gel), glass, and elastomer (PDMS). First, the BAC‐gel is polymerized atop a through‐hole glass slide, creating a stable, covalently bonded trapdoor. After the BAC‐gel polymerizes, the Bis‐gel is then polymerized directly on top of the BAC‐gel, forming the microwells with the BAC‐gel as a base of the microwell (Figure S1, Supporting Information). This layered assembly allows for efficient isolation of intact mammalian cells for downstream applications, such as perturbation, imaging, or proteomics analysis. The through‐hole glass slide acts as a gate for isolating the nucleus from each cell, providing structural support throughout the single‐cell handling steps, and ensuring stability during the alignment to the PDMS nucleus‐receiving microwells. Without the support of the glass, the thin composite of BAC and Bis‐gels (≈100 µm thick) would collapse during the dissolution process. Additionally, the through‐hole glass slide forms a well‐defined path for nuclei to travel from the Bis‐gel microwells, through the trapdoor, and into the PDMS microwells. This setup physically transfers each nucleus into a PDMS compartment compatible with standard biochemical processes, such as PCR. Glass is an ideal material for this design due to its strong, stable bonding properties with both polyacrylamide and PDMS, which are commonly used in single‐cell and single‐molecule analyses.

The trapdoor at the base of each top‐layer Bis‐gel microwell is designed to be initially closed, to open with chemical and mechanical triggers, and then remain open (Figures 1E,2A,B). To achieve these functions, the trapdoor is composed of a layer of *N*, *N*’‐bis(acryloyl)cystamine (BAC), a reversible crosslinker, polymerized with acrylamide monomer to form a dissolvable PAG layer (BAC‐gel), cast on a 400‐µm thick glass slide with laser‐etched 100‐µm diameter through‐holes (Figure 2A; Figure S1, Supporting Information).^[^ ^39^ ^]^ Application of DTT results in the degradation of the disulfide‐cross‐linked BAC‐gel due to the thiol–disulfide exchange reaction (Figure 2D).^[^ ^40^, ^41^, ^42^ ^]^ Application of a suction force to the bottom of the PDMS microwell receiving layer transfers force up to the sandwiched trapdoors and initiates nucleus transfer from the Bis‐gel microwells into said PDMS receiving microwells. To be effective at transmitting the suction force from the bottom of the PDMS microwells to the trapdoors, the receiving PDMS microwells are designed with ultra‐thin (≈40 µm) bases (floors). With the vacuum manifold mated to the bottom of the multi‐layer assembly, the PDMS microwell floor flexes outward upon application of suction and material is pulled – via the trapdoor – from the top Bis‐gel microwell into the receiving PDMS microwell (Figure 1E).

### 2.2 Alignment Strategy for Fabrication of the Multi‐Layered, Interconnected VacTrap

For nucleus transfer to be successful, each 40‐µm diameter top‐layer Bis‐gel microwell must be polymerized and aligned atop of each 100‐µm diameter glass through‐hole coated with a 50‐µm thick BAC‐gel layer that will function as a trapdoor conduit to the receiving 250 by 350‐µm PDMS microwell (Figure S1, Supporting Information). The BAC‐gel here will act as a temporary base of the Bis‐gel microwell. Alignment must be achieved to sufficient precision across the 15 by 15 mm, 256 Bis‐gel microwell array. One time‐sensitive constraint arises: How to delay the polymerization and formation process of the Bis‐gel microwell until the microwell's location is defined to align with each through‐hole of the glass slide?

To achieve this balance of timing, we implemented two distinct polymerization methods for BAC‐gel and Bis‐gel, each having a different time constant for polymerization. Since there is no restriction to the location or polymerization time of the BAC‐gel, we employed chemical polymerization for the BAC‐gel layer. The BAC‐gel was polymerized on top of the through‐hole glass slide using acrylamide monomers and BAC as a cross‐linker through free radical polymerization with tetramethylethylenediamine (TEMED) and ammonium persulfate (APS). To precisely position the Bis‐gel microwells directly over the BAC‐gel‐coated through‐holes (Figure S1, Supporting Information)—ensuring the effective transfer of nuclei across multiple layers of the VacTrap system—the Bis‐gel was photopolymerized using 2,2‐Azobis[2‐methyl‐N‐(2‐hydroxyethyl)pro‐pionamide] (VA‐086, 1%) as a photoinitiator.^[^ ^43^ ^]^ A photomask was employed to define the microwell diameter and location. Under UV exposure, the Bis‐gel precursor in the transparent regions of the mask was exposed and polymerized, while the opaque regions (containing 256 circular features, each 40 µm in diameter) blocked UV, preventing polymerization to form the microwells (Figure S1, Supporting Information). This photopolymerization approach allowed sufficient time to align the mask with the through‐holes, ensuring the Bis‐gel microwells were accurately positioned over the BAC‐gel‐coated through‐holes, forming a composite gel. To initiate nucleus transfer, the composite gel was then aligned to the PDMS microwell and the vacuum manifold using brightfield microscopy. Our vacuum manifold utilizes trapezoid pillars (surrounding each PDMS microwell) to prevent the PDMS microwell from collapsing when a vacuum force is applied (Figure 2C). This configuration ensures continued contact between the through‐hole glass slide and the PDMS microwell.

### 2.3 Design and Fabrication of the Trapdoor Features

The diameter of the top‐layer whole‐cell receiving Bis‐gel microwells is designed to closely match the diameter of individual mammalian cells (≈30–40 µm). To achieve an aspect ratio (1.3) designed to reduce the likelihood of capturing multiple mammalian cells in each microwell, we fabricate 60‐µm tall Bis‐gel microwells.^[^ ^33^ ^]^

To enhance the cell‐settling efficiency, the Bis‐gel whole‐cell receiving layer is dehydrated prior to introducing a cell suspension. Drying polyacrylamide microwell results in the microwell diameter expanding upon dehydration by ≈1.5× for gels chemically polymerized (e.g., TEMED, APS). Deviations from an aspect ratio of ≈1.3 lead to >1 cell per microwell occupancy, which is not desired in single‐cell resolution assays or sample preparation. Consequently, for a photopolymerization (vs chemical polymerization) process, we sought to understand the effect of UV dose (energy × exposure duration) on photopolymerization of the Bis‐gel atop the dissolvable BAC‐gel layer.

We asked what range of UV doses minimize Bis‐gel expansion after dehydration, while preserving a target hydrated Bis‐gel microwell diameter of 40 µm. All the while, the process maintains the Bis‐gel layer as co‐planar on top of the polymerized dissolvable BAC‐gel in such a way that 1) the BAC‐gel fully covers the top of the glass through‐holes and 2) the Bis‐gel microwells are each aligned with the through‐holes in the glass slide (Figure 1B; Figure S2A, Supporting Information). Across a wide UV‐dose range (1400–2000 mJ cm^−^^2^), we measured a ≈1.5× expansion in diameter for the Bis‐gel microwells after dehydration when polymerizing using the lowest UV doses (1400 and 1600 mJ cm^−^^2^) (Figure S2, Supporting Information). At 1400 mJ cm^−^^2^, we observed darkening beneath the microwells by brightfield microscopy, particularly when approaching the through‐hole glass slide during a *z*‐axis sweep (Figure S2B, Supporting Information). We attributed the observation to potential under‐polymerization of the Bis‐gel, as indicated by a 57% increase in diameter after dehydration (*∅*_hydrated_ = 37.2 ± 3.5 µm, *∅*_dehydrated_ = 58.6 ± 5 µm, *N* = 100) (Figure S2C, Supporting Information). In contrast, a higher UV dose of 2000 mJ cm^−^^2^ resulted in a 25% shrinkage of the microwell diameter, with both hydrated and dehydrated microwells being too small for single‐cell encapsulation (*∅*_hydrated_ = 31.7 ± 3.1 µm, *∅*_dehydrated_ = 26.1 ± 2.8 µm, *N* = 100). Optimal results were achieved at a UV dose of 1700 mJ cm^−^^2^, where the diameter of 100 microwells was measured at *∅*_hydrated_ = 35.3 ± 1.8 µm and *∅*_dehydrated_ = 46.1 ± 3.3 µm, thus maintaining the target microwell diameter of ≈32 and 40 µm before and after drying, respectively, as is suitable for single mammalian cell encapsulation.

### 2.4 Despite the Partial Expansion of Bis‐Gel Microwells upon Dehydration, the VacTrap Workflow Remains Unaffected

We designed the system such that, even with a potential 30% increase in Bis‐gel microwell diameter (resulting in a maximum of ≈46 µm, as shown in Figure S2, Supporting Information), the Bis‐gel microwells remain significantly smaller than the ≈100 µm through‐hole trapdoor and the 250 × 350 µm PDMS microwells. This size differential provides substantial tolerance for minor variations. Consequently, the Bis‐gel microwells still align reliably over the through‐holes, and subsequently, the through‐holes align with the larger PDMS microwells. Thus, minor fluctuations in Bis‐gel microwell dimensions do not compromise VacTrap's overall alignment or functionality (Figures S2,S3, Supporting Information). Based on the observations on the expansion of the Bis‐gel microwell, we posit that increasing the UV dose increases the Bis‐gel stiffness and, thus, reduces the susceptibility of Bis‐gel microwells to expansion upon dehydration. Previous research by Sheth et al.^[^ ^44^ ^]^ determined that the Young's modulus – a measure of the stiffness of a hydrogel – is directly proportional to UV dose. The study considered photopolymerization of polyacrylamide hydrogels with the photoinitator Irgacure 2959 across a UV‐dose range of 1500–2600 mJ cm^−^^2^. The proposed underlying mechanism implicates a higher UV dose to enhanced crosslinking reactions, resulting in the formation of more functional crosslinks in the resultant gel versus those observed in a lower UV dose process. The crosslinks increase gel stiffness and, for our purposes, make the hydrogel less likely to shrink upon dehydration.

### 2.5 Chemico‐Mechanical Actuation of Trapdoors to Open the Fluidic Connection between Stacked Microwell Layers

We next sought to identify chemical and mechanical conditions well suited to actuating the physical transfer of a nucleus through each trapdoor feature. Previous research has reported 10–20 mm DTT dissolving 0.392–0.500% BAC‐gels in 1–5 min.^[^ ^42^, ^45^ ^]^ However, these previous studies have considered dissolution of BAC‐gel in a bulk form or as BAC‐gel droplets immersed in a DTT solution, with thermoshaking.^[^ ^42^, ^45^ ^]^ Our layered microfluidic device presents a materially different dissolution environment for DTT‐actuated BAC‐gel dissolution. In our layered system, DTT must diffuse from the point of application, through a 60‐µm‐deep Bis‐gel microwell, and then dissolve the 50‐µm‐thick BAC‐gel layer from the top.

In tandem with considerations of the BAC‐gel composition, we considered compatible approaches to apply force to the dissolving BAC‐gel and expedite formation of a fluidic interconnection between the two layers. Primary among our considerations was a vacuum‐driven force wherein we attached a microfluidic vacuum manifold underneath the nucleus‐receiving PDMS microwell layer. PDMS casting fabricated a thin PDMS floor in each nuclei‐receiving PDMS microwell (thickness ≈40 µm). The thin PDMS floor is important to provide physical compliance sufficient to effectively transfer vacuum‐generated suction force from the vacuum manifold layer to the contents of the PDMS microwell, the BAC‐gel in the glass through‐holes, and finally into the upper Bis‐gel microwell compartment. The vacuum manifold generates continuous suction across the gas‐permeable PDMS thin floor, allowing air to flow from the Bis‐gel microwell through the BAC‐gel and glass through‐holes, which in turn drives DTT flow through the Bis‐gel microwell, efficiently dissolving the BAC‐gel.

To prevent the collapse of the PDMS microwell and floor when vacuum force is applied, we employed an array of structural‐support pillars surrounding each PDMS microwell, thereby ensuring supportive structural contact between the through‐hole glass slide and the PDMS microwell layer (**Figure** **3**A). We first employed circular cross‐section pillars and observed physical behavior and features when the BAC‐gel layer incorporated a fluorescently labeled acrylamide monomer via fluorescence microscopy. However, we found that the circular cross‐section pillars did not prevent PDMS microwell deformation under application of the vacuum force (Figure S4, Supporting Information). Circular cross‐section pillars (total surface area = 0.00785 mm^2^) resulted in detachment between the PDMS microwell and the vacuum manifold. In contrast, when the larger‐surface‐area trapezoidal cross‐section pillars (total surface area = 0.2418 mm^2^) were employed, the PDMS microwell was not observed to either deform or detach, and fluidic connectivity was observed between the stacked Bis‐gel and PDMS microwells (Figure 3A,B). Consequently, we opted to utilize trapezoidal cross‐section support pillars around the PDMS microwells.

To understand the importance of not just dissolving the BAC‐gel comprising the trapdoor but also applying a gentle suction force on that depolymerized BAC‐gel bolus, we observed the dissolution process with and without applied vacuum from the vacuum manifold (Figure 3B) for a BAC‐gel trapdoor fabricated with 0.2% BAC crosslinker, using 40 mm DTT. With the vacuum applied, dissolution through a 50‐µm‐thick BAC‐gel layer occurred within 10 min (Figure 3B, Video S1, Supporting Information). Upon activation of the vacuum, the DTT rapidly reached the microwell, indicated by the reduction in fluorescence signal from the BAC‐gel on top of the through‐hole glass. Within the subsequent 5 min, dissolution commenced. Dissolution was considered complete when the fluorescence signal from the BAC‐gel reached its maximum before a reduction in fluorescence. The signal trend indicates that the dissolved fluorescent BAC‐gel initially accumulates at the glass through‐holes, resulting in a peak fluorescence signal. As the gel continues to dissolve, the liquified gel passes through the through‐hole and into the underlying PDMS microwell, causing a decrease in fluorescence as the material moves out of the focal plane. With the vacuum support, the BAC‐gel around the through‐holes is fully dissolved, indicated by the loss of fluorescence in the through‐hole area (Figure 3C). In contrast, when a trapdoor feature composed of 0.2% BAC was exposed to 40 mM DTT without application of an external mechanical force (‐ vacuum), the BAC‐gel layer did not dissolve and transfer into the PDMS microwell, and no fluidic nor materials connection was observed between the stacked Bis‐gel and PDMS microwells after 30 min (Figure 3B, Video S2, Supporting Information), as evidenced by the fluorescence signal of BAC‐gel remaining around the through‐hole (Figure 3C). The vacuum facilitated dissolution in an average of 80% of microwells (≈179 microwells). Without applied vacuum, dissolution of the BAC‐gel was observed in less than 5% of microwells (Figure 3D). Dissolution efficiency depends on the precise alignment of the Bis‐gel microwell and trapdoor feature with the lower‐layer PDMS microwell to ensure the suction force is transmitted effectively through the microwell stack. Ensuring the timely dissolution of the BAC‐gel is crucial to maintaining the integrity of the Bis‐gel microwell. Without specific dissolution within the through‐hole area only, the Bis‐gel can detach due to a loss of structural support from the BAC‐gel and the through‐hole glass slide (Figure 3C). These observations suggest the significance of applying suction to facilitate fluidic interconnection between the stacked microwell layers.

To understand the practical implications of dissolving a BAC‐gel in a layered configuration, we studied parameters that influence the dissolution rate, including BAC concentration, DTT concentration, and UV dose used in Bis‐gel photopolymerization (Figure 3E–G). We first tested a range of BAC crosslinker concentrations from 0.150% to 0.400%. Higher BAC concentrations resulted in a stiffer gel exhibiting a longer time to dissolve. Therefore, we aimed for a low BAC concentration to facilitate rapid dissolution of the BAC‐gel layer while still maintaining the integrity of the Bis‐gel microwell and dissolvable trapdoor feature. We observed that a 50‐µm thick BAC‐gel with 0.150% BAC can be dissolved by 100 mm DTT in 3 min (Figure 3E), which was in the target dissolution‐performance range. However, this lower BAC concentration made the gel more susceptible to tearing during the fabrication process. With that in mind, a 0.200% BAC was observed to dissolve within 3–5 min using 100 mm DTT (Figure 3E), still within the desired timeframe but with enhanced mechanical robustness which is helpful for reliable fabrication. Taken together, a 0.2% BAC‐gel was selected for further analysis.

In tandem, we considered a range of DTT concentrations from 20 to 100 mm for dissolution of trapdoor features fabricated with 0.2% BAC‐gel, with complete dissolution achieved in 3 min with 100 mm DTT. Application of 20 mm of DTT required ≈15 min for dissolution (Figure 3F). However, DTT is a common redox reagent used to break down protein disulfide bonds, including antibodies.^[^ ^46^ ^]^ We sought to minimize its concentration for downstream proteomics applications via scWB in the Bis‐gel microwell layer. We selected 40 mm DTT, followed by multiple washes with high‐pH buffer (>8) at elevated temperatures to deactivate residual DTT before immunoprobing.

DTT does not interfere with PCR or reverse transcription, making this dissolvable gel suitable for common genomic and nucleic acids applications such as DNA or RNA‐seq.^[^ ^42^ ^]^ We also considered alternative reducing agents, including TCEP, β‐mercaptoethanol (BME), and photocleavable crosslinkers for the trapdoor. However, BME, despite being a weaker reducing agent, is highly toxic and prone to rapid oxidation upon air exposure,^[^ ^47^ ^]^ making it less desirable for our workflow. Photocleavable crosslinkers were also ruled out as they require UV exposure for cleavage,^[^ ^48^ ^]^ which would prematurely disrupt the trapdoor before the completion of the scWB workflow and nucleus transfer. While TCEP presents an alternative, it is a highly reactive reducing agent that indiscriminately cleaves disulfide bonds, including those critical for protein structure and antibody recognition,^[^ ^49^, ^50^ ^]^ which is not suitable for our VacTrap workflow including proteomics analysis. Given these limitations and DTT's well‐established use in RNA‐seq protocols, we determined that DTT provided the best balance of reducing strength, compatibility, and reliability for our system. Thus, considering these factors, we opted to use DTT in our workflow.

We found that the UV dose used for Bis‐gel photopolymerization did affect the dissolution of the underlying BAC‐gel trapdoor, with increasing UV dose increasing the required dissolution time (Figure 3G). However, choosing a low dose of UV for Bis‐gel photopolymerization could lead to underexposure causing microwell expansion and incomplete polymerization beneath the Bis‐gel microwell (Figure S2B, Supporting Information). We hypothesize that UV‐based activation homolytically cleaves disulfide bonds to yield two separated thiol radicals.^[^ ^51^, ^52^ ^]^ While disulfide bonds could reform if the radical species generated remain in proximity after cleavage, the radicals may recombine with different thiol radicals within the gel matrix, not necessarily from the same original disulfide bridge. Such recombination would cause an observed temporal delay in dissolution. Moreover, excess photoinitiator (VA‐86) trapped in the Bis‐gel may lead to further polymerization of the BAC‐gel around the microwell area, causing further delay in dissolution. With 100 mm of DTT, a 0.2% BAC concentration, and a 1700 mJ cm^−2^ UV exposure for the Bis‐gel, dissolution was completed in <5 min without any detectable damage to the Bis‐gel microwell after dissolution.

### 2.6 Actuation of Trapdoor Features Allows Concurrent Physical Transfer of Isolated Nuclei

**Figure 4:**
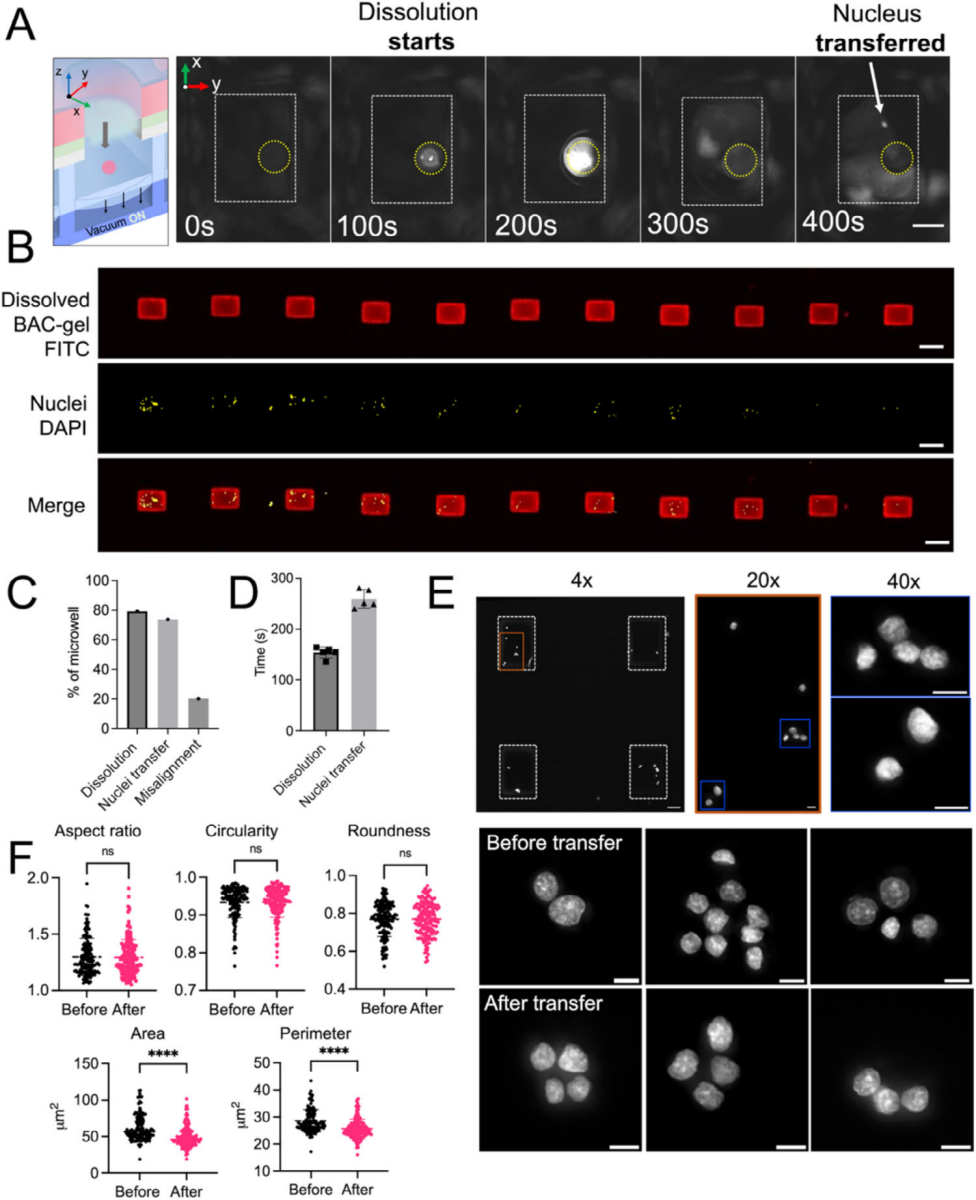
VacTrap simultaneously transfers nuclei across an array of stacked microwells while maintaining nuclear morphological integrity. A) Time‐lapse fluorescence micrographs showing transfer of isolated nuclei from breast cancer cell line cells (MCF‐7) through the BAC‐gel trap door into a PDMS microwell. For visualization, nuclei stained with HOECHST, settled into the cell‐receiving Bis‐gel microwells. The BAC‐gel trapdoor was FITC‐acrylate labeled and opened (dissolved) with 100 mm DTT followed by vacuum activation. Scale bar: 100 µm. B) False‐colored fluorescence micrographs of a row of PDMS microwells after applying the VacTrap system demonstrate the transfer of dissolved BAC‐gel trap door (red) and nuclei (yellow, DAPI) into PDMS microwells. For nuclei visualization, nuclei were HOECHST stained, and ≈20 000 nuclei were settled into the cell‐receiving Bis‐gel microwells before washing with PBS to remove excess nuclei. To visualize the trap doors opening, FITC‐acrylate‐labeled was added to the BAC‐gel trapdoor, which was dissolved with 100 mm DTT, followed by vacuum activation to mobilize the BAC‐gel bolus. Scale bar: 250 µm. From top to bottom: Dissolved trapdoor of BAC‐gel (red), transferred nuclei (yellow), and merged micrographs. C) Microscopy‐based analysis of nucleus transfer yield shows the percentage of microwells (%) showing both trapdoor dissolution and successful nucleus transfer are nearly identical, with discrepancies attributed to misalignment between the nucleus‐receiving PDMS microwell on the bottom layer and the whole‐cell receiving Bis‐gel microwells on the top device layer. D) Dissolution time and nucleus transfer across 6 representative trapdoor features suggests nearly synchronized trapdoor actuation across the microwell array. E) Fluorescence microscopy inspection of transferred nuclei housed in the nucleus‐receiving PDMS microwell array suggests nuclei remain intact after transfer. Scale bar: 100 µm (4×) and 10 µm (20× and 40×). F) Morphological analysis of transferred nuclei before and after trapdoor‐assisted transfer that relies on a combination of trapdoor dissolution (DTT) and applied suction using the integrated vacuum manifold layer (ns: non‐significant, **** *p*‐value ≤ 0.0001).

We sought to understand the capability of VacTrap to transfer isolated nuclei through the dissolved BAC‐gel while maintaining the physical integrity of the nucleus after an applied (suction) mechanical force. To assess simple physical integrity of isolated nuclei, we employed fluorescence microscopy to inspect whether transferred nuclei were physically intact or physically compromised after transfer through a 0.2% BAC‐gel trapdoor dissolved by applying 100 mm DTT and suction. **Figure** **4**A and Video S3 (Supporting Information) illustrate nucleus transfer through the BAC‐gel trapdoor into the nuclei‐receiving PDMS microwell. Nuclei were observed transferring into the nucleus‐receiving PDMS microwells at ≈360 s after vacuum activation while the dissolution began at ≈135 s (Video S3, Supporting Information). By fluorescently labeling both the BAC‐gel in the trapdoor feature and the isolated nuclei with HOECHST 33342 we observed nuclei transferred into ≈80% of PDMS microwells inspected (Figure 4B,C).

To understand the degree of synchronization in the dissolution times across the trapdoor features in a microwell array (Figure 4D), we monitored dissolution with fluorescence microscopy and measured trapdoor BAC‐gel dissolution times ranging from 136–160 s, with an average of 154 ± 10 s (*N* = 6) with nucleus transfer occurring at ≈105 ± 15 s post‐dissolution. The observed delay between the initial dissolution of the BAC‐gel trapdoor and nucleus transfer arises from the requirement for complete dissolution of the BAC‐gel, which requires 3–5 min with 100 mm DTT. Ensuring simultaneous dissolution and transfer is essential for maintaining the integrity of the nuclei throughout the entire microwell array.

To assess the overall yield of trapdoors with suitable performance, inspection of the microwells by microscopy during BAC‐gel dissolution revealed that ≈80% of the BAC‐gel microwells dissolved, corresponding with the percentage of nucleus transferred within the same microwell array (Figure 4D). The high but not perfect yield in functional trapdoor features is attributed to misalignment between the stacked pair of Bis‐gel and PDMS microwell arrays. Additionally, PDMS is known to shrink when cured at high temperatures such as those used in this study, so curing PDMS microwells at room temperature should reduce shrinkage and enhance alignment accuracy.

Finally, to extend understanding beyond the physical integrity of extracted nuclei, we sought to assess nuclear phenotype (e.g., morphology). Here, we leveraged the transparency of the PDMS microwells to assess the morphology of nuclei before and after transfer (Figure 4E,F). Common morphological parameters including nucleus aspect ratio, circularity, roughness, area, and perimeter were analyzed (Figure 4F). Our results indicate no detectable changes in aspect ratio, circularity, or roughness before and after nucleus transfer. However, alterations in area and perimeter were observed. We hypothesize that the changes in area and perimeter are attributable to the response of nuclei upon exposure to DTT during the transfer process as well as imaging artifacts that arise from imaging through the PDMS microwells. Nevertheless, nuclei remained intact and retained their overall shape.

### 2.7 Simultaneous Single‐Cell Protein Immunoblotting and Nucleus Isolation

We next aimed to evaluate the complete VacTrap workflow, which begins with single‐cell settling and proceeds through cytoplasmic lysis using DDF, protein preservation via PAGE, nucleus transfer, and immunoprobing for target proteins. A key consideration in this process is ensuring that the cytoplasmic lysis buffer and electrophoresis step do not compromise nuclear integrity. Previously, we demonstrated that our cytoplasmic buffer selectively lyses the cytoplasm while leaving the nucleus intact, as shown by bidirectional scWB^[^ ^34^ ^]^ and PCR.^[^ ^38^ ^]^ However, neither scWB nor PCR alone fully captures the chromatin state which defines the nuclear architecture.

**Figure 5:**
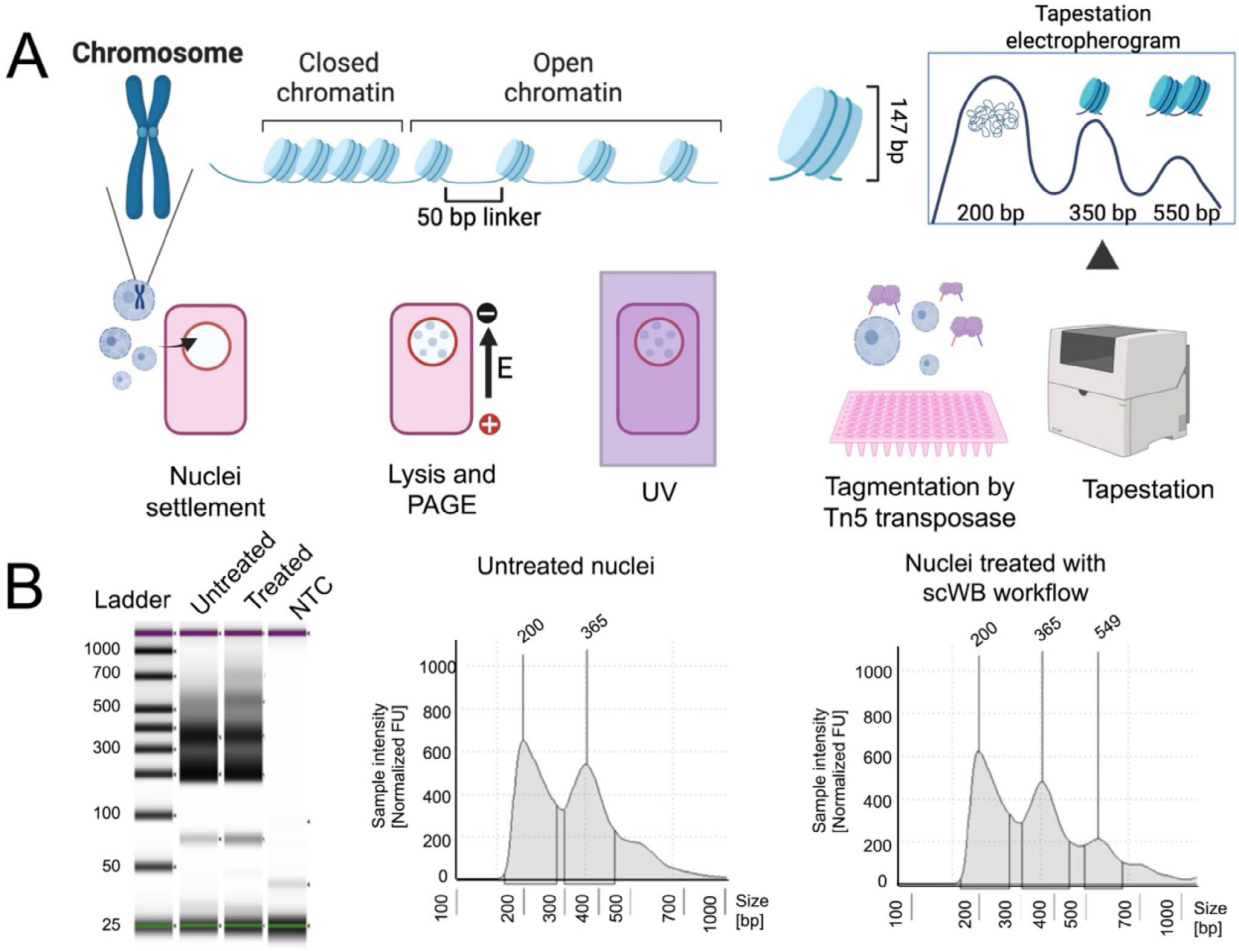
Assessment of nucleus quality by nucleosomal pattern analysis following exposure to the single‐cell western blot (scWB) workflow, which comprises DDF lysis buffer, electrophoresis (PAGE), and UV‐based protein immobilization (labeled as “Treated”). A) Schematic illustrating the workflow and nucleosome structure, chromatin states, and TapeStation fragment analysis. In eukaryotes, chromatin consists of DNA (≈147 bp) wrapped around histone octamers to form nucleosomes, separated by 50 bp linker DNA. Here, ≈20 000 nuclei were settled into silicone pad microwells and subjected to cell cytoplasmic lysis buffer, PAGE, and UV‐based protein immobilization, then recovered for tagmentation with Tn5 transposase. If nuclei, thus chromatin structure, remain intact, characteristic peaks appear at ≈200 bp (open chromatin), ≈350 bp (mononucleosome), and ≈550 bp (dinucleosome). Fragmented nuclei would lose these higher base pair (bp) nucleosomal peaks. B) TapeStation DNA electrophoresis (left) and nucleosomal patterns analysis (right) show comparable profiles among isolated nuclei exposed to lysis, PAGE, and UV‐based immobilization (“Treated”), relative to untreated control labeled as “Untreated”. The TapeStation electropherogram confirms peaks corresponding to open chromatin (≈200 bp), mononucleosomes (≈350 bp), and dinucleosomes (≈550 bp), indicating that nucleus integrity is largely preserved throughout the scWB process. NTC = negative template control.

To investigate chromatin structure more directly, we employed the assay for transposase‐accessible chromatin using the hyperactive Tn5 transposase to access the regions of open chromatin. This process reveals chromatin accessibility and nucleosomal organization: intact nuclei typically yield three characteristic fragment peaks (≈200 bp for open chromatin, ≈350 bp for mononucleosomes, and ≈550 bp for dinucleosomes). In our VacTrap experiments, isolated MCF7 nuclei were exposed to the subcellular scWB workflow steps—including cytoplasmic lysis buffer, the electric field during PAGE, and UV‐based protein immobilization—before being recovered for any downstream analysis (**Figure** **5**A). As a control, we analyzed nuclei that did not undergo the procedure. The Tapestation profiles (Figure 5B) revealed no notable differences between the exposed nuclei and untreated controls, indicating that the VacTrap process does not disrupt chromatin structure. These results confirm that our protocol preserves nuclear architecture, as assessed by chromatin accessibility analysis.

Last, we sought to understand the capacity of the system to fractionate and preserve each cell's cytoplasmic proteome from each intact nucleus. To answer this fractionation and preservation question, we introduced two capabilities to allow us to monitor and detect cytoplasmic protein markers during the VacTrap process of single‐cell nuclear fractionation. First, to assess fractionation and preservation of the cytoplasmic proteome in real time during the VacTrap process, we chose to assay a cell line engineered to express a naturally fluorescent protein (tGFP) in the cytoplasm of each cell. The engineered version of the human malignant glioblastoma multiforme (GBM) U251 cell line is referred to here as U251‐tGFP. Second, we added a performance diagnostic step to the VacTrap process by integrating a single‐cell western blot to verify isolation and preservation of the cytoplasmic proteome of each cell. To add this verification function, the Bis‐gel layer was modified to perform single‐cell western blotting for direct detection of non‐fluorescent cytoplasmic proteomic targets by antibody‐based probing. Taken together, the scrutiny of a naturally fluorescent cytoplasmic protein (tGFP) and adaptation of the Bis‐gel layer to perform single‐cell western blotting allows direct detection of cytoplasmic protein markers as verification of the fractionation and, ultimately, the preservation of each cell's cytoplasmic proteome.

**Figure 6:**
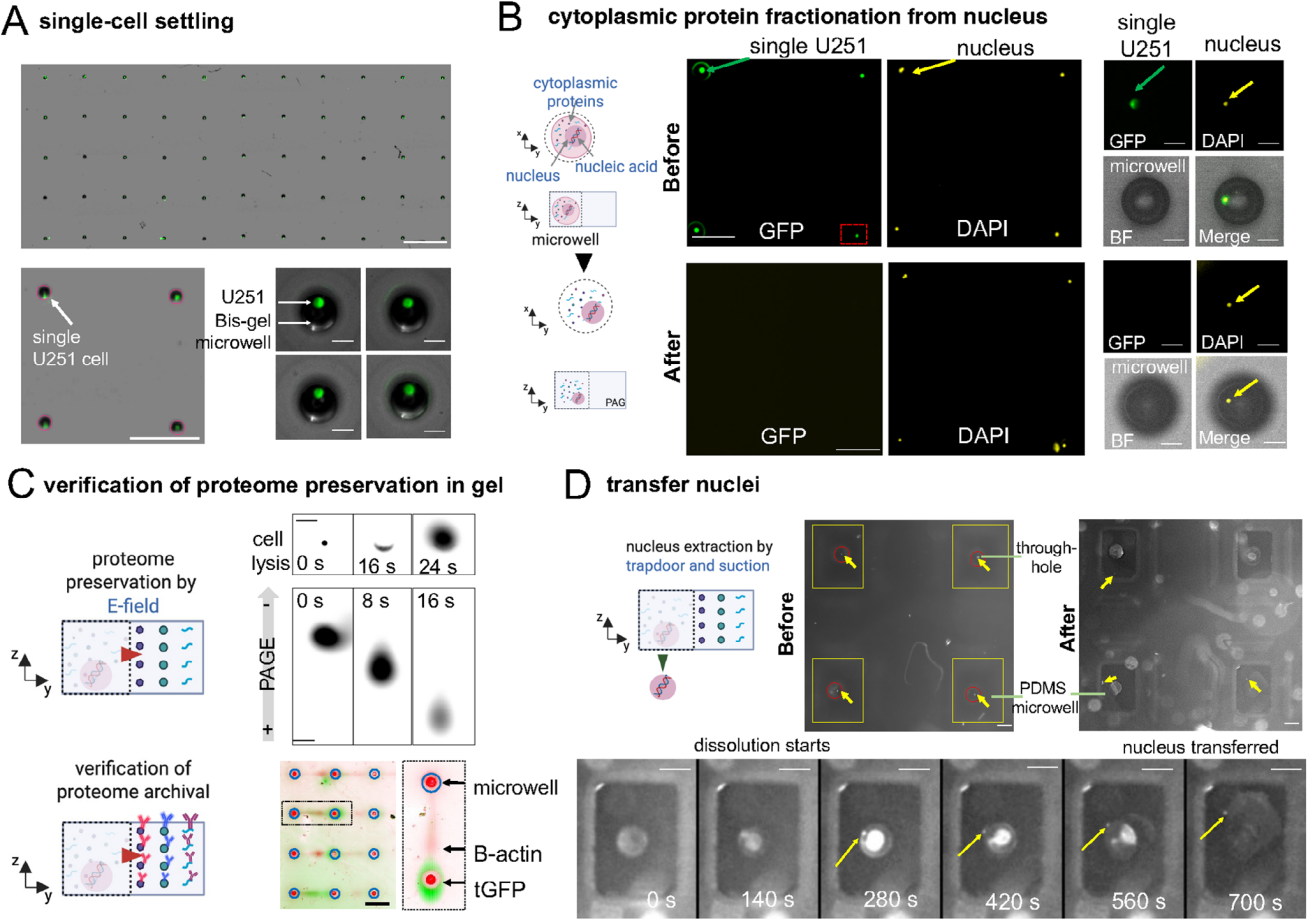
Single‐cell western blotting confirms fractionation of the cytoplasmic proteome from each nucleus for individual U251‐tGFP glioblastoma cells. A) Widefield fluorescence micrograph of single U251‐tGFP cells isolated in an array of Bis‐gel microwells. Close‐up view of four microwells, each housing a single U251‐tGFP cell. False‐color green fluorescence signal is from tGFP expression. Scale bars: 1 mm widefield; 500 µm close up; 40 µm individual microwell. B) Fluorescence micrographs of microwell‐isolated U251‐tGFP cells during fractionation of the cytoplasmic proteome from the nucleus. Micrographs before (top) and after (bottom) proteomic fractionation by i) cytoplasmic lysis using DDF (30 s, room temperature) and ii) proteome extraction by PAGE (40 s at 40 V cm^−1^). False‐color fluorescence is tGFP and DAPI nucleus staining. Nuclei are verified as physically intact (yellow arrows) after the proteome fractionation process. Scale bars: 250 µm widefield; 20 µm close up. C) False‐color micrographs of the proteome fractionation process (top) and verification (bottom) by immunoprobing. (Top) Time‐lapse micrographs show the process of cytoplasmic lysis (cell lysis) by DDF and proteome extraction by PAGE. (Bottom) Single‐point micrographs verify the isolation of tGFP (intrinsic green fluorescence) and beta‐actin (immunoprobing using beta‐actin antibody probe) from each microwell. False‐color green signal is tGFP; red signal is immunoprobed beta‐actin. Scale bar: 60 µm time‐lapse images; 500 µm endpoint image. D) Endpoint fluorescence micrograph of microwell array before and after nucleus transfer through a trapdoor and into a PDMS microwell (bottom layer) from a Bis‐gel microwell (top layer). Corresponds to the four cells shown in (B). The left panel shows nuclei isolated in microwells before transfer (yellow arrows). The right panel shows successfully transferred nuclei (yellow arrows). The time‐lapse sequence (bottom row) reports the dissolution of the BAC gel trapdoor with DTT and physical transfer of each nucleus and dissolved BAC gel trapdoor from the Bis‐gel microwell into the corresponding PDMS microwell. Applied suction accelerates the physical transfer of each nucleus – and encapsulates bolus of BAC gel – from the Bis‐gel microwell into the PDMS microwell. Corresponds to the single cell inset shown in (B). After cytoplasmic proteome fractionation, the Bis‐gel was washed in PBS for 15 min, briefly dried, cleaned on the backside, and aligned with the PDMS microwell on the vacuum manifold. Then, 200 µL of 40 mm DTT was applied on top of the gel for 2 min, followed by vacuum actuation. Dissolution began at 140 s, with nucleus transfer completed by 700 s. Scale bar: 100 µm.

Recall that the VacTrap multi‐step workflow comprises: U251 single‐cell settling, DDF‐mediated cytoplasmic lysis, protein electrophoresis, nucleus transfer, composite‐gel separation, and immunoblotting. U251 cells were first settled into Bis‐gel microwells (**Figure** **6**A) before cytoplasmic lysis and PAGE. As shown in Figure 6B, the nuclei remained intact in the microwells throughout DDF cytoplasmic lysis, PAGE, and UV exposure, evidenced by the persistent nuclear fluorescence both before and after lysis, while the U251 cytoplasmic tGFP signal was absent from the microwell after lysis. During cytoplasmic lysis, TurboGFP was tracked for 30 s in DDF and then for 40 s during electrophoresis at 40 V cm^−1^ in the Bis‐gel (Figure 6C, Video S4, Supporting Information). During electrophoresis, tGFP migrated similarly as observed previously in standard subcellular single‐cell western blots.^[^ ^34^ ^]^ Cytoplasmic proteins were then photocaptured in the Bis‐gel and archived for subsequent immunoblotting following nuclear transfer (Figure 6C).

Next, we aligned the composite gel (containing nuclei in Bis‐gel microwells) with PDMS microwells featuring a trapezoid‐pillar vacuum manifold (Figure 6D). The nuclei were retained in the Bis‐gel microwells atop the BAC‐gel trapdoor prior to transfer (Figure 6D). After ≈140 s of exposure to 40 mm DTT and vacuum suction force, the gel began to dissolve, and by ≈280 s, the nuclei started transferring into the PDMS microwells via interconnected liquid channels. Complete transfer was achieved in 700 s (Videos S5,S6, Supporting Information). When starting from single cells rather than isolated nuclei, transfer was slightly delayed, likely due to the electric field driving nuclei to the microwell periphery and causing nuclei to adhere to the edges.

Last, immunoblotting of the composite gel detected B‐actin alongside photocaptured TurboGFP (Figure 6C), confirming that the VacTrap system supports integrated archiving of the cytoplasmic proteome after nucleus extraction from each single cell.

## 3 Conclusion

In this study, we introduced the VacTrap system, a multilayer microfluidic device designed to facilitate high throughput, spatially indexed transfer of nuclei from each cell of hundreds of single cells, with proteomics analysis via single‐cell western blot. By integrating a stacked pair of microwells – a top layer that is the cell‐receiving Bis‐gel microwell array and a bottom layer that is the nucleus‐receiving PDMS microwell array – with interleaving dissolvable trapdoor features and a vacuum‐driven force actuation system, VacTrap simultaneously extracts and transfers isolated nuclei across hundreds of microwells and single cells within 3–5 min. Importantly, VacTrap preserves nuclear integrity and indexing of each nucleus back to its originating cell and any associated data collected on that intact cell prior to nuclear extraction.

As a sample preparation device, VacTrap automates the functions of isolating and measuring (e.g., imaging) individual intact cells, fractionation of the nucleus from each imaged cell, and then synchronizes the physical transfer of the isolated nuclei into a PDMS microwell array suitable for subsequent nuclei measurement and analysis (e.g., PCR). To provide a compartment for intact cell imaging and nuclei fractionation, and one for chemical manipulation of isolated nuclei, VacTrap is designed with a stacked microwell array comprising: 1) a top‐layer of Bis‐gel microwells used to isolate the originating single cell, and 2) a bottom‐layer of PDMS microwells used to compartmentalize each extracted nucleus. During cytoplasmic lysis, the nucleus has been fractionated from the originating cell, while the proteomics content is archived via PAGE into the Bis‐gel layer. The VacTrap system establishes a fluidic connection on demand between the stacked microwell pair using chemical and mechanical actuation to transfer the nuclei. Spatial indexing of hundreds of originating intact cells to their resultant fractionated nuclei and proteins takes advantage of the microarray layout, which is compatible with time‐lapse imaging. A precision single‐cell preparation technique, VacTrap enhances the throughput of organelle isolation (here, demonstrated for nuclei) and ensures the rapid and reliable transfer necessary for downstream multi‐omics analyses, thus demonstrating potential for inclusion in single‐cell multi‐omics research tools.

Applied here to two mammalian‐cell case studies – breast cancer (MCF7, 20–30 µm diameter) and glioblastoma (U251, 100–200 µm diameter when in spheroid form) cell lines – the VacTrap framework is designed to be adaptable to diverse cell and tissue types. By adjusting the microwell diameter (while maintaining an aspect ratio near 1.3), previous work has reported on single‐cell microwell‐array isolation for a wide variety of cell lines and primary cells, including neuronal stem cells, HEK293, MCF7, SKBR3, and MDA‐MB‐231, as well as patient‐derived circulating tumor cells, dissociated solid breast tumors, and single murine blastomeres, and pre‐implantation embryos up to blastocysts.^[^ ^36^, ^53^, ^54^ ^]^ Tailoring lysis buffer composition and detergent strength can further ensure selective permeabilization across different membrane rigidities or unique cellular architectures (e.g., non‐mammalian systems). As such, the fundamental trapdoor mechanism described here is neither restricted to a particular cell size nor buffer condition; rather, it comprises a tunable design that can be optimized for biological contexts. Future efforts may explore specialized chemical formulations or microwell geometries suitable for plant cells, yeast, or other challenging specimens, broadening VacTrap's applicability while retaining single‐cell resolution and high‐throughput indexing capabilities.

## 4 Experimental Section

### Chemicals

Tetramethylethylenediamine (TEMED, T9281), 40% Acrylamide solution (A4058), Acrylamide/Bis‐acrylamide 40% solution (29:1), *N*, *N*’‐Bis(acryloyl)cystamine (A4929), Ammonium persulfate (APS, A3678), Methanol (34 860), Dimethylsulfoxide (DMSO, D2438), Fluoresceine O Acrylate (568 856), and 3‐(Trimethoxysily)propyl methacrylate were obtained from Sigma Aldrich.

Dithioerythritol (DTT) solution (0.1 m), phosphate‐buffered saline (PBS, 10 010 023), and Hoechst 33342 solution (20 mm, 62 249) were purchased from Thermo Fisher. BP‐APMA (BPMAC) was custom‐synthesized by Raybow. Photoinitiator 2,2‐Azobis(2‐methyl‐N‐(2‐hydroxyethyl)propionamide) (VA‐086) was acquired from FujiFilm Wako Pure Chemical Corporation. Gel Slick was purchased from Lonza (#50 640). Molecular biology grade water was from Corning (46‐000‐CV). Tris‐glycine (10×) buffer (25 mm Tris, pH 8.3; 192 mm glycine) was obtained from Bio‐Rad (#1 610 734). Methacryloxyethyl thiocarbamoyl rhodamine B (Rhodamine B methacrylate, 23591‐100) was purchased from Polysciences. IGEPAL CA‐630 MegaPure Detergent, 10% solution was acquired from Abcam (ab285400). 10% Tween‐20 Dnase/Rnase Tested, Sterile was from Teknova (Teknova T0027). Digitonin solution supplied at 20 mg mL^−1^ in DMSO was purchased from Promega (G9441).

### Buffers

Nuclei were isolated using ATAC‐RSB buffer^[^ ^55^ ^]^ which was prepared by mixing 500 µL of 1 m Tris‐HCl pH 7.4 (10 mm), 100 µL of 5 m NaCl (10 mm), 150 µL of 1 m MgCl_2_ (3 mm), and 49.25 mL of molecular biology grade water. The lysis buffer was prepared by adding 0.1% IGEPAL CA‐630, 0.1% Tween‐20, and 0.01% digitonin to the ATAC‐RSB buffer to reach the final volume. The wash buffer contained 0.1% Tween‐20 in ATAC‐RSB buffer.

### Fabrication of the Trapdoor BAC‐Gel Layer on Through‐Hole Glass Slides

Hundred‐micrometer‐diameter, 400‐µm thick through‐hole glass slides with slide dimensions of 28 by 40 mm, were generously provided by Arralyze (LPKF Laser and Electronics AG, Germany). BOROFLOAT 33 (Schott AG), a borosilicate glass that was widely used for life science applications due to low autofluorescence and high optical transparency in the visible region, was used as a substrate. Details on the Laser Induced Deep Etching protocol can be found in previous studies, and was used in through‐hole fabrication by ARRALYZE.^[^ ^39^ ^]^ The slides were silanized to enhance the gel bonding on the glass slide by adding a methacrylate group as previously described.^[^ ^33^ ^]^

BAC‐gel fabrication was performed in a glove bag (Thermo Scientific, 09 3737.LK) with continuous nitrogen flow to prevent oxygen inhibition of polymerization. Kapton tape rails (50‐µm; Digikey, 315‐CQT‐0.250‐ND) were used to set the BAC gel height. Two rails were taped onto a large glass slide (Ted Pella, 260234‐25) with 20 mm spacing to align the through‐hole area. The glass slide was washed with IPA and dried with nitrogen. Gel slick^R^ (Lonza,600 µL) was spread between the Kapton tape rails and dried at room temperature. The glass slide was then rinsed with water and dried with a Kimwipe using a buffing motion to remove excess gel slick.

A 20 mm × 18 mm PDMS membrane was cut and applied to one side of the through‐hole glass slide to limit gel precursor diffusion into the through‐holes during BAC‐gel fabrication. The through‐hole glass slide was taped tightly atop the 50‐µm height Kapton tape rails on the large glass slide with the PDMS membrane facing up. At least ≈5 mm from each long side of the through‐hole glass slide should sit on top of the Kapton tape rails, resulting in gel‐free edges after fabrication. The assembly was degassed for 10 min before being moved to a glove bag until the gel precursor was ready.

### BAC Gel Precursor

10% APS (w/v) and 10% TEMED (v/v) were prepared with molecular biology grade water and moved to the glove bag. BAC solution was made by dissolving ≈22 mg of BAC in 100% methanol, followed by vortexing. BAC‐gel precursor was prepared with 6% (w/v) acrylamide, 1× Tris‐Glycine (pH 8.3), molecular grade water, and various concentrations of BAC (0.150%, 0.200%, 0.250%, 0.350%, and 0.400%). For some experiments, 100 mm fluorescein o‐acrylate in DMSO was added to the gel precursor for a final concentration of 0.2 mm. The gel precursor was degassed and sonicated for 15 min before adding 10% APS and 10% TEMED at a final concentration of 0.1% under the glove bag. Gel precursor (1 mL) was quickly wicked through the through‐hole glass slide and polymerized under nitrogen for 20 min. After polymerization, the through‐hole glass slides and the PDMS membrane were removed, and the gel was incubated with DI water for 5 min before removal from the rails. The gels were kept in water for at least 2 h before use.

### Fabrication of the Bis‐Gel Microwells with Photopolymerization

A customized 8 × 8 photomask (Artnet Pro) with 40‐µm‐diameter dark circular features and transparent fields was affixed to heat‐resistant borosilicate glass (8″ × 6″, 1/8″ thickness, McMaster Carr 8476K72). Two pieces of 60‐µm‐thick Kapton tape (3m 5419) were applied on top of the photomask to form two rails for Bis‐gel fabrication and to determine the Bis‐gel height, set to cover the through‐holes and BAC‐gel area. The area between the two rails served as a wicking area for Bis‐gel precursor.

Gel slick (400 µL) was applied between the rails and dried at room temperature for 3 min. Excess gel slick on the mask was cleaned with a Kimwipe using a circular buffing motion. On the other side of the glass plate, a long pass filter sheet (8″ × 6″) was cut and fixed with Kapton tape.

The BAC‐gel was dried with nitrogen before attaching a 20 mm × 18 mm PDMS membrane to cover the through‐hole. Two pieces of 50‐µm Kapton tape were applied to the gel‐free edges (≈5 mm wide) of the through‐hole glass slide and cut to shape. These rails compensated for BAC‐gel height expansion when exposed to the Bis‐gel precursor. Then the BAC‐glass was aligned to the photomask so that the through‐hole could be seen aligned with the opaque circular spot on the photomask defining where the Bis‐gel microwell would be and aligned to the through‐hole. Kapton tape was used to affix the glass on the mask before. The entire assembly was moved to a vacuum chamber and kept closed without a vacuum until the gel precursor was ready.

VA‐086 photoinitiator was dissolved in water to a final concentration of 2% (w/v). Bis‐gel precursor was prepared with molecular‐grade water to a final concentration of7% Acrylamide/Bis‐acrylamide (29:1), 3 mm BPMAC in DMSO, 1× Tris‐glycine (pH 8.3), and 1% VA‐086, adjusted with molecular biology grade water. To stain the gel, 100 mm Rhodamine B methacrylate was added to the precursor for a final concentration of 0.2 mm. The gel precursor was degassed for 10 min before wicking through the BAC‐gel through‐hole glass slide and vacuuming until all bubbles were removed.

The glass plate was then placed under an OAI UV exposure system (Optical Associates, Incorporated) with UV power of ≈20 mW cm^−^^2^ (OAI UV Probe 365 nm, measured without the long‐pass filter) for doses of 1400, 1600, 1700, 1800, and 2000 mJ cm^−^^2^. The standard dose for most experiments was 1700 mJ cm^−^^2^ (≈85 s exposure with ≈20 mW cm^−^^2^ UV energy measured without the long‐pass filter). After photopolymerization, gels were incubated with water for 5 min before detachment. The composite gels were kept in molecular biology‐grade water until use.

### Soft Lithography for Fabrication of PDMS Layers

The PDMS microwell array consisted of 16 rows by 16 columns of rectangular microwells, each measuring 350 µm by 250 µm, with a 1‐mm spacing center‐to‐center. The microwell SU‐8 mastermold was fabricated using SU‐8 2100 (Kayaku Advanced Materials) to achieve a height of 200 µm, following the manufacturer's instructions. Then the PDMS microwells were produced by spinning ≈5 g of a 10:1 PDMS mixture on the SU‐8 mastermold in two steps: the first step for 5 s at 100 rpm with an acceleration time of 5 s, and the second step for 30 s at 400 rpm with an acceleration of 100 rpm, followed by 3 h curing at 80 °C. Before any experiment, the PDMS microwell was deposited in the air plasma cleaner (PDC‐32G, Harris plasma) with a vacuum setup of 0.470 torr using an ICME vacuum pump. The radiofrequency power was set to High for 3 min.

A vacuum manifold was prepared by casting ≈27 g of a 10:1 PDMS mixture onto a 100 µm height SU‐8 mold using SU‐8 3050 (Kayaku Advanced Materials), also according to the manufacturer's instructions. The vacuum manifold featured trapezoid structures with bases of 250 and 443 µm, and legs of 147 µm. Prior to PDMS casting, the PDMS mixture was degassed for 1 h before being applied to the wafers. All PDMS was cured at 80 °C for 3 h and allowed to cool to room temperature before use. The vacuum manifold had four outlets, which were created using a 2.5 mm biopsy punch (Integra) and connected to soft PVC Plastic Tubing for Air and Water, 1/32″ ID, 3/32″ OD (McMaster‐Carr) for vacuum application.

### Cell Lines

Cancer cell lines MCF7 Tet‐off parental cells (RRID: CVCL_V357) and U251 (RRID: CVCL_0021) were used throughout this study. All cell lines were authenticated by short tandem repeat (STR) profiling at the UC Berkeley Cell Culture Facility and tested negative for mycoplasma, confirming that they were contamination‐free. The MCF7 Tet‐off parental cells were generously provided by the Arribas Lab at the Vall d'Hebron Institute of Oncology.

### Nucleus Isolation

For each experiment, MCF7 Tet‐off parental cells were maintained at 37 °C and 5% CO_2_ in Dulbecco's Modified Eagle Medium/Nutrient Mixture F‐12 (Gibco DMEM/F‐12, GlutaMAX supplement, Thermofisher, 10 565 018) supplied with 10% fetal bovine serum (Gemini Bio), 0.2 mg mL^−1^ Gibco Geneticin Selective Antibiotic (G418 Sulfate), and 1 µg mL^−1^ doxycycline (Sigma) until 80% confluency and detached with 0.05% Trypsin‐EDTA (Gibco #25300‐054) for 4–5 min.

One million viable cells were aliquoted into 1.5 mL LoBind Eppendorf tubes. The cells were then centrifuged at 500 g for 5 min at 4 °C. After centrifugation, the medium was removed, and the cells were resuspended in 1 mL of cold 1× PBS buffer. The cells were centrifuged again at 500 g for 5 min at 4 °C, and the PBS was aspirated. Subsequently, 300 µL of cold lysis buffer was added to the sample, and the cells were mixed 10 times. The sample was incubated on ice for 5 min. After incubation, 1 mL of cold wash buffer was added to each sample, and the tubes were inverted 5 times to mix. The nuclei were pelleted with the hinge facing in at 500 g for 3 min at 4 °C, then centrifuged again with the hinge facing out at 500 g for 3 min at 4 °C. The supernatant was aspirated in two steps: 1000 µL was removed with a P1000 pipette, and the remaining 50–100 µL was removed with a P200 pipette. The nuclei were gently resuspended in 250 µL of wash buffer using a wide‐bore tip (Rainin). The quality and count of the nuclei were assessed using a Countess automated cell counter (10 µL of nuclei with 10 µL of Trypan blue)

To fluorescently label nuclei for imaging, 2 µL of 20 mm Hoechst was added into 1000 µL of PBS to prepare the staining wash buffer. An aliquot of 100,000 nuclei was added to 1000 µL of staining wash buffer and incubated for 20 min on ice. The nuclei were pelleted with the hinge facing in at 500 x g for 5 min at 4°C, then centrifuged again with the hinge facing out at 500 x g for 5 min at 4 °C. The supernatant was aspirated, and the nuclei were resuspended in 1000 µL of PBS to achieve a concentration of ≈100 nuclei µL^−1^.

### Alignment and Assembly of the VacTrap Device Layers

Before aligning the device, the composite gel was gently dried using nitrogen. The back of the glass slide was then cleaned with Scotch tape to ensure a seamless contact between the PDMS microwell and the composite gel. The composite gel was initially aligned with the PDMS microwell, then inverted, and the vacuum manifold was carefully applied to the back of the PDMS microwell.

### Actuation of the Bis‐Gel Trapdoor Features

The four outlets of the vacuum manifold were connected to tubing and a house vacuum system. Subsequently, 300 µL of DTT was delicately applied to the surface of the gel, followed by a 2‐minute incubation period before activating the vacuum.

To assess nucleus transfer between the composite gel and PDMS microwell arrays, ≈20,000 HOECHST‐labeled nuclei were allowed to gently settle onto the cell‐receiving Bis‐gel microwells for 10–15 min. Afterward, the gel was washed with PBS to remove excess nuclei. FITC‐acrylate‐labeled BAC‐gel trapdoor was activated with 100 mm DTT, followed by vacuum activation. Time‐lapse imaging was conducted using a 4× PLAN APO objective, capturing multiple nuclei within each microwell.

Nucleosomal pattern validation using tagmentation by Tn5 transposase after nuclei exposing to cytoplasmic buffer and an electrophoresis step. To form a chamber for isolated nuclei to undergo the complete scWB workflow and assess nuclear integrity prior to the VacTrap protocol, nucleosomal pattern analysis by Tn5 tagmentation was performed. A silicone adhesive pad (1‐mm thick, 20 × 70 mm) was punched with 10 holes using a 2 mm biopsy punch (Integra) and affixed onto a glass slide. Nuclei were isolated as described above. Following two rounds of centrifugation (3 min each), the nuclei pellet was resuspended in 250 µL of wash buffer. Next, 10 µL of the nuclei suspension was dispensed into each well of the silicone pad, and the nuclei were allowed to settle for 15 min.

Subsequently, the glass slide was mounted in the electrophoresis chamber and subjected to the scWB protocol.^[^ ^33^ ^]^ Briefly, 15 mL of cytoplasmic buffer was overlaid on the slide for 30 s, followed by 20 s of electrophoresis and 45 s of UV exposure. After these steps, the glass slide was removed, and nuclei were collected from each well by pipetting. The nuclei were then centrifuged at 500 × g for 5 min at 4 °C, washed in PBS, and resuspended to a final concentration of 300 nuclei µL^−1^. Samples were maintained on ice until use. A control nuclei solution was similarly prepared using the standard nucleus isolation protocol, diluted to 300 nuclei µL^−1^, and kept on ice.

To verify nucleus integrity after cytoplasmic lysis, electric field application during PAGE, and UV exposure during protein immobilization, the tagmentation method was employed using Tn5 transposase to evaluate the nucleic acid integrity after the workflow. A tagmentation cocktail was prepared using 1× Tagmentation buffer (Illumina Inc), 0.1% Tween 20, and 0.01% digitonin (Promega). TDE1 tagmentation enzyme (Tn5, Illumina Inc) was added to the cocktail at a ratio of 1:15. Then, 1.5 µL of nuclei was aliquoted into a mini 96‐well plate (Kubo Biotech), followed by the addition of 2.5 µL of the tagmentation mix. The plate was sealed with PCR tape and incubated for 30 min at 37 °C in a qPCR instrument (q225, Kubo Biotech).

To terminate the tagmentation reaction, 2 µL of stop buffer (0.025 m EDTA, 0.015 m Tris‐HCl, pH 8.0) was added to each well, and the mixture was incubated for 30 min at 50 °C. For library amplification, 9 µL of PCR mix containing 1× NEBNext High‐Fidelity 2× PCR Master Mix, 1.25 µm i5 primer (5′‐AATGATACGGCGACCACCGAGATCTACAC[i5]TCGTCGGCAGCGTC‐3′), and 1.25 µm i7 primer (5′‐CAAGCAGAAGACGGCATACGAGAT[i7]GTCTCGTGGGCTCGG‐3′) was added to each well. The amplification protocol was as follows: 72 °C for 5 min; 98 °C for 30 s; then 10 cycles of 98 °C for 10 s, 63 °C for 30 s, and 72 °C for 60 s. PCR products were purified using the Zymo DNA Clean and Concentrator‐5 kit. DNA concentration was quantified using a Qubit fluorometer and dsDNA high sensitivity assay kit, and the nucleosomal pattern was assessed using an Agilent TapeStation with D1000 tape and corresponding reagents.

### Single Cell Protein Analysis in the Composite Gel

U251 cells were stably transduced with TurboGFP via lentiviral infection (multiplicity of infection 10). U251 cells were cultured in DMEM, high glucose, GlutaMAX Supplement (10566‐016) with 1% penicillin/streptomycin (15 140 122, Life Technologies), BenchMark FBS (100–106, Gemini Bio‐Products), 1× MEM nonessential amino acids (11 140 050, Life Technologies), and 1 mm sodium pyruvate (11360‐070) in an incubator at 37 °C with humidified 5% CO_2_ air. The composite gel was dehydrated using a nitrogen stream. U251 cells engineered to express cytoplasmic TurboGFP were gravity settled in microwells by pipetting 400 µL of cells in PBS at a concentration of 1.5 × 10^6^ cells mL^−1^. Live whole‐cell nuclei were labeled as described previously. Cells gravity settled for 15 min, and unsettled cells were washed away by placing the gel at a 45° angle and gently flowing 15 mL of PBS on the surface of the gel to remove unsettled cells. The gel was taped with double‐sided Kapton tape onto the clear electrophoresis chamber for recording the timelapse. The cytoplasmic buffer was composed of 1%v/v Triton X‐100 (Sigma Aldrich X100‐100ML), 0.124 mg mL^−1^ Digitonin (Sigma Aldrich D141‐500), and 0.5× Tris‐Glycine (BioRad 1 610 734). The electrophoresis chamber was fabricated by laser cutting acrylic sheets, supergluing the acrylic sheets onto the edges of a petri dish while leaving a 3 cm space between acrylic sheets, and taping platinum electrodes 3 cm apart onto the petri dish using Kapton tape. Fractional cell lysis was performed for 30 s at room temperature and electrophoresed for 40 s at 40 V cm^−1^. Photocapture of protein was accomplished with a UV source (100% power, 45 s, Lightningcure LC5, Hamamatsu). Gels were kept in PBS for 15 min until nucleus transfer with VacTrap as described previously with 40 mm of DTT. Then the entire system was spun down at 2000 RPM for 2 min. Then the composite gel was washed in Tris‐HCl pH 8.5 for 1 h at 37 °C with buffer exchange every 15 min. The composite gel was stored in 1× TBST overnight at 4 °C, and then it was immunoprobed as previously described.^[^ ^33^ ^]^ The primary antibody was incubated for 2 h with 40 µL of antibody cocktail and washed in 1× TBST for 1 h (buffer exchanged every 30 min). The primary antibody cocktail for the multimodal measurements was rhodamine labelled anti‐Beta‐Actin (1:5 dilution, 12 004 163) in 2% BSA in 1X TBST.

### Imaging

All imaging reported in this study was performed using an Olympus IX51 microscope with various Plan Apo objectives (4×, 10×, 20×, and 40×) and filter sets for GFP, Texas Red, and UV (DAPI). Confocal imaging was conducted with a Bruker Confocal Microscope at the UC Berkeley QB3 Cell and Tissue Analysis Facility, utilizing an Olympus Plan APO 40× water immersion objective. Additionally, some imaging in this study was performed using a Genepix MicroArray Scanner (Genepix 4300A, Molecular Devices). Image processing was done using ImageJ, and nuclei morphology was analyzed with the MicrobeJ plugin.

## Conflict of Interest

The authors declare no conflict of interest.

## Supporting information



Supporting Information

Supplemental Video 1

Supplemental Video 2

Supplemental Video 3

Supplemental Video 4

Supplemental Video 5

Supplemental Video 6

## Data Availability

The data that support the findings of this study are available from the corresponding author upon reasonable request.
