# Supplementary material for: Single‐Cell Nucleus Extraction with Cellular Indexing
Source: Adv Sci (Weinh). 2025 Sep 29;12(46):e14883. doi: 10.1002/advs.202514883 (PMC12697769; doi:10.1002/advs.202514883)
Supplement: Supplementary file 1 — Supporting Information [file ADVS-12-e14883-s006.docx]

**SUPPLEMENTAL FIGURES**

**Single-cell Nucleus Extraction with Cellular Indexing**

*Trinh Lam, Ana E. Gomez Martinez, Alison Su, Anna Fomitcheva-Khartchenko, Xin Wang, Md Nazibul Islam, Paul Lum, and Amy E. Herr**

T. Lam, A.E. Gomez Martinez, A. Su, A. Fomitcheva-Khartchenko, X. Wang, Md N. Islam, P. Lum, and Prof Amy E. Herr

Department of Bioengineering

University of California, Berkeley

Berkeley, CA 94720, USA

Prof Amy E. Herr

Chan Zuckerberg Biohub San Francisco

San Francisco, CA 94158, USA

Email: aeh@berkeley.edu

***Keywords:*** single-cell, organelle, multiomics, hydrogel, proteomics, and microfluidics

**Supplemental Figures**

**Figure S1.** Schematic of the BAC-gel and Bis-gel fabrication and dissolution process. The 50 μm height BAC-gel is chemically polymerized onto a through-hole glass slide within a nitrogen glove bag, followed by the fabrication of the Bis-gel atop the BAC-gel using photopolymerization employing a photomask and UV exposure. BAC-gel dissolution is initiated by the application of DTT and vacuum suction force.

**Figure S2. Effect of UV exposure dose on fabrication of the Bis‑gel microwell array for single cell isolation.** For visualization, Bis-gel was co-polymerized with 0.2 mM Rhodamine B methacrylate while BAC-gel was co-polymerized with 0.2 mM fluorescein-o-acrylate (FITC acrylate). (**A**) Fluorescence micrographs of the whole-cell receiving Bis-gel microwell array layer (red) and BAC-gel-on-glass trapdoor features (blue), along with merged image. Scale bar: 1 mm. (**B**) Characterization of hydrated and dehydrated Bis-gel microwell features photopolymerized under a range of 360-nm UV doses. Dehydration results in a slight expansion of features, as expected. Scale bar: 50 μm. (**C**) Diameter of resultant Bis-gel microwells fabricated using a range of UV doses, for hydrated and dehydrated imaging conditions (N=100 microwells for each condition). The diameter of the hydrated and dehydrated microwells were closest to the desired 40 μm diameter at a UV dose of 1700 mJ/cm², for this formulation.


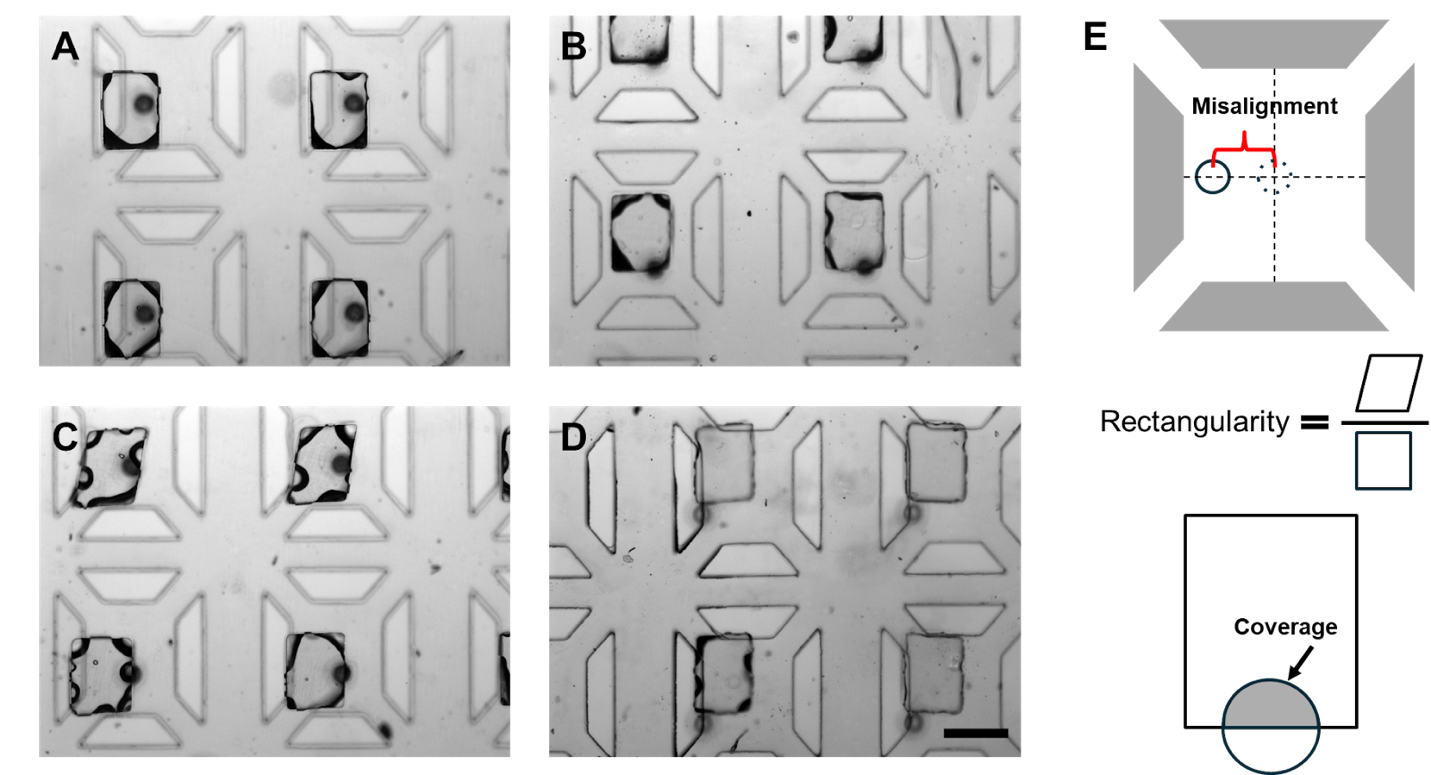


**Figure S3.** Robustness of the VacTrap system alignment. (A) The VacTrap system successfully transfers content from the polyacrylamide microwell to the PDMS well despite a polyacrylamide microwell misalignment of up to 52% and a PDMS well misalignment of up to 78% (measured as center-to-center displacement). (B) Content transfer is achieved even when the polyacrylamide microwell is only partially covered by the PDMS well, with successful transfer observed at 60% coverage. (C) The system remains effective even when the PDMS microwell loses its rectangular shape due to stretching during alignment, as demonstrated by successful transfer at a rectangularity value of 1.12 (where 1 represents a perfect rectangle). (D) Example of unsuccessful content transfer due to severe misalignment between layers. (E) Schematic representation of the quantification methods for misalignment, coverage, and rectangularity. Scale bar: 400 µm

**Figure S4.** Fluorescence micrographs of pre- and post-actuation of the trapdoor feature by an applied suction force levied by the vacuum manifold with circular pillars. For visualization, the BAC-gel is copolymerized with 0.2 mM FITC acrylate in all fluorescence images reported in this Figure. Fluorescence micrographs show deformation and detachment of circular structural pillars after suction is applied to the PDMS microwell by the vacuum manifold. Scale bar: 100 µm.


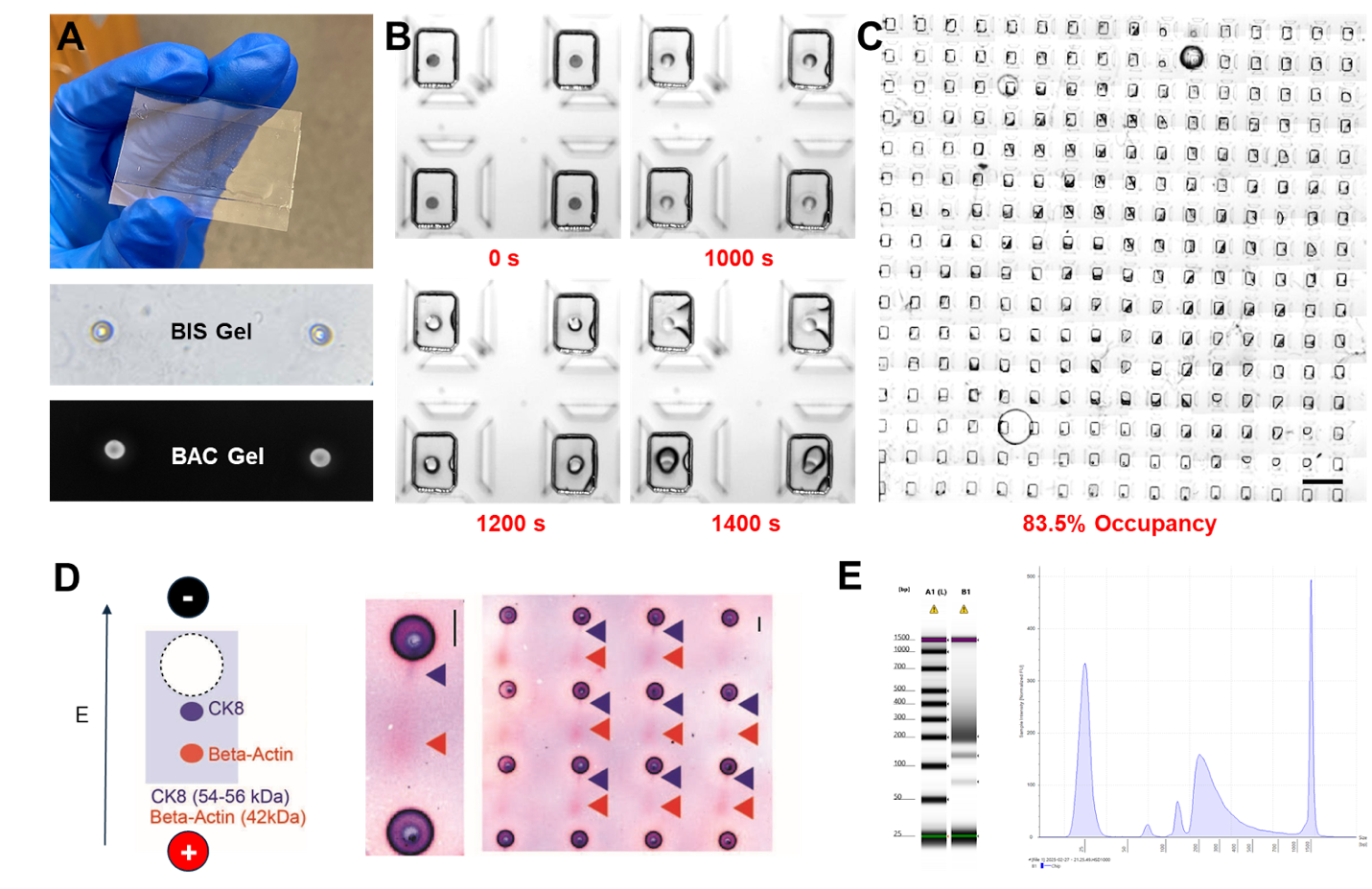


**Figure S5.** **Replicability of VacTrap fabrication and operation by a second generation of independent researchers.** (A) Fabrication of the composite gel containing a BAC gel trapdoor and a Bis-gel microwell atop a through-hole glass slide. (B) Time-lapse images showing the dissolution of the BAC layer upon treatment with DTT under vacuum. (C) Demonstration of successful content transfer from polyacrylamide microwells to PDMS microwells via BAC layer dissolution, achieving an 83.5% occupancy rate. (D) Single-cell Western blot performed on the fabricated composite gel, confirming the detection of two proteins, CK8 (54–56 kDa) and B-Actin (42 kDa). (E) TapeStation quality control analysis confirming the successful transfer of nuclear content into the PDMS well. Scale bar: 1 mm
